# Biocompatible Liquid Embolic for the Treatment of Microvascular Hemorrhage

**DOI:** 10.1002/advs.202403615

**Published:** 2024-07-25

**Authors:** Hyeongseop Keum, Jinjoo Kim, Zefu Zhang, Erin Graf, Hassan Albadawi, Rahmi Oklu

**Affiliations:** ^1^ The Laboratory for Patient‐Inspired Engineering, Mayo Clinic 13400 East Shea Blvd Scottsdale AZ 85259 USA; ^2^ Department of Laboratory Medicine and Pathology, Mayo Clinic 5777 E Mayo Blvd Phoenix AZ 85054 USA; ^3^ Division of Vascular & Interventional Radiology, Mayo Clinic 5777 E Mayo Blvd Phoenix AZ 85054 USA

**Keywords:** angiography, coagulopathy, embolization, hemorrhage, microvessels

## Abstract

Persistent or recurrent bleeding from microvessels inaccessible for direct endovascular intervention is a major problem in medicine today. Here, an innovative catheter‐directed liquid embolic (P‐LE) is bioengineered for rapid microvessel embolization to treat small vessel hemorrhage. Tested in rodent, porcine, and canine animal models under normal and coagulopathic conditions, P‐LE outperformed clinically used embolic materials in both survival and non‐survival experiments, effectively occluding vessels as small as 40 microns with no signs of recanalization. P‐LE occlusion is independent of the coagulation cascade, and its resistance to displacement is ≈ 8 times greater than systolic blood pressure. P‐LE is also found to be biocompatible and x‐ray visible and does not require polymerization or a chemical reaction to embolize. To simulate the clinical scenario, acute microvascular hemorrhage is created in the pig kidney, liver, or stomach; these are successfully treated with P‐LE achieving immediate hemostasis. Furthermore, P‐LE is found to be bactericidal to highly resistant patient‐derived bacteria, suggesting that P‐LE may also protect against infectious complications that may occur following embolization procedures. P‐LE is safe, easy to use, and effective in treating ‐microvessel hemorrhage.

## Graphical abstract

An innovative catheter‐directed liquid embolic (P‐LE) is bioengineered to achieve rapid and durable segmental hemostasis reaching microvessels. This novel approach demonstrated remarkable efficacy in rat, pig (non‐survival and survival), and in anti‐coagulated dog embolization models, outperforming the clinically used embolic agents. P‐LE represents a next‐generation liquid embolic; it is biocompatible, x‐ray visible, bactericidal, and embolizes distal, often unreachable microvessels with the potential to improve patient outcomes and potentially save lives.

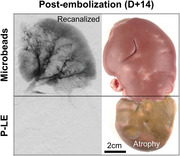

## 1 Introduction

Minimally invasive endovascular angiography, employing techniques such as coil, bead, or gel embolization, is increasingly preferred for managing acute vascular hemorrhage.^[^ ^1^ ^]^ This approach involves a pin‐hole percutaneous entry through the common femoral artery and enabling a catheter to be guided to the site of the bleeding vessel. Metallic coils or Obsidio gel embolic materials are then introduced through the catheter and positioned proximal to the bleeding site to achieve hemostasis.^[^ ^2^ ^]^ In bead‐based embolization, spherical particles are released into the vessel; these particles are carried by the blood flow to smaller branches where they accumulate, effectively occluding the vessel based on the size of individual beads or bead clusters. Both metallic coils and beads rely on the body's ability to form a thrombus around them to occlude the vessel; if the patient is coagulopathic, bleeding will likely persist. In scenarios of re‐bleeding or breakthrough bleeding post‐embolization, the risk of mortality increases tenfold.^[^ ^3^ ^]^

**Figure 1:**
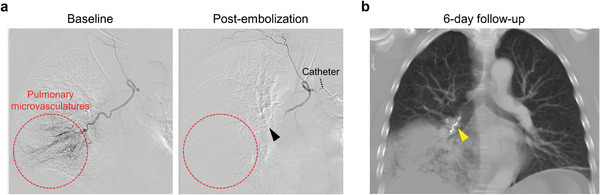
Limited access of Obsidio™ conformable gel embolic to the distal microvessels. a) Digital subtraction angiography (DSA) in a 63‐year‐old male with acute and severe hemoptysis secondary to lung adenocarcinoma; red circle indicates the area of hemorrhage. The patient underwent bronchial arterial embolization using Obsidio gel embolic, resulting in instant hemostasis (black arrow; red circle). The gel embolic successfully embolized the upstream larger bronchial artery and did not address the smaller bleeding blood vessels distally. b) The computed tomography (CT) chest angiogram at 6‐day follow‐up revealed a stable Obsidio gel embolic (yellow arrow) but confirmed that the peripheral microvasculatures were not directly targeted. A few days after embolization, bleeding resumed from the distal microvessels eventually requiring an elective surgical resection.

Microvascular hemorrhage can be seen in patients with chronic inflammation or infections, such as cystic fibrosis or tuberculosis, leading to hemoptysis; neoplastic conditions or minor trauma are other causes of microvascular hemorrhage, especially in patients who are on anticoagulants.^[^ ^4^ ^]^ In these cases, the strategy for treatment often involves embolizing accessible upstream blood vessels. By occluding these larger, upstream vessels, it is anticipated that the flow to the hemorrhaging microvessels will be interrupted, effectively treating the bleed. This approach is taken because directly targeting the numerous and tiny sub‐millimeter vessels is not feasible (**Figure** **1**). To overcome the limitations of coils, beads, and Obsidio embolization techniques, clinicians occasionally resort to the off‐label use of two FDA‐approved polymer‐based liquid embolic agents, Onyx, which is composed of ethylene vinyl alcohol, and Trufill, made from n‐butyl cyanoacrylate. Despite their potential, the liquid embolic agents Onyx and Trufill present significant drawbacks. Clinical trials have shown that 77.3% of patients injected with Onyx experienced neurological issues, such as visual impairment and ataxia, while gastrointestinal (nausea, vomiting, heartburn) and vascular complications (hematoma, bleeding) were observed in 47% and 43.9% of patients, respectively.^[^ ^5^ ^]^ In early 2012, the FDA issued a safety alert about the risk of cementing the catheter when using Onyx, with 54 reported cases of catheter adhesion leading to surgical interventions and, in some instances, fatalities. Onyx is also associated with a 20% rate of incomplete occlusion and a 12–36% risk of recanalization, along with procedural risks like rupture and leakage during injection.^[^ ^6^ ^]^ Trufill carries its own risks, including non‐target embolization, delayed or premature polymerization leading to migration or catheter blockage.^[^ ^7^ ^]^ Additionally, most liquid embolics require the use of dimethyl sulfoxide (DMSO) to prevent in‐catheter polymerization, necessitating costly DMSO‐compatible catheters.^[^ ^8^ ^]^ DMSO itself is toxic and linked to vasospasm, angionecrosis, and transient pulmonary complications, as it is partly exhaled through the lungs.^[^ ^9^ ^]^ These drawbacks limit the widespread adoption of current DMSO‐based liquid embolic agents as effective alternatives to coil, gel, or bead embolics in treating microvascular hemorrhage. An ideal embolic agent should be able to reach the smallest distal microvasculature for quick, more effective hemostasis, regardless of a patient's anticoagulation status, while also being biocompatible and easy to prepare for efficient and immediate use.

**Figure 2:**
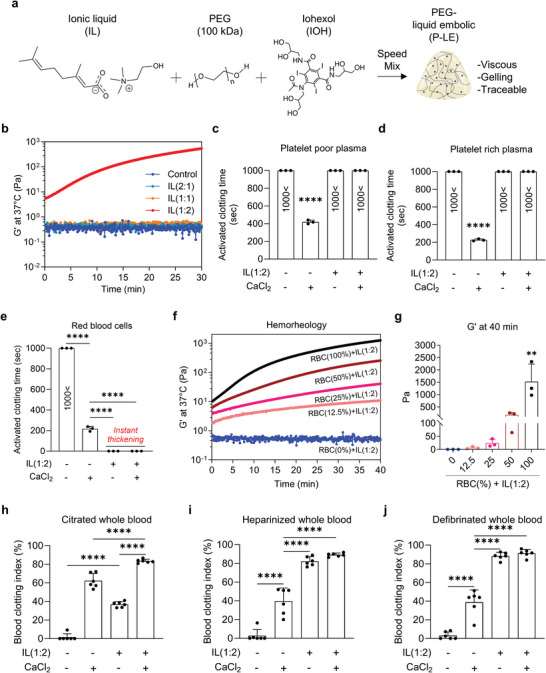
Characterization and mechanism of P‐LE. a) The formulation of P‐LE consists of IL, PEG (100 kPa), and IOH. b) Hemorheology curves of blood mixed with IL generated in different stoichiometric ratios. c–e) Activated clotting time (ACT) of platelet‐poor plasma c), platelet‐rich plasma d), and red blood cells e), when mixed with IL (−/+) or CaCl_2_ (−/+) (n = 3). f) Representative hemorheology curves of various concentrations of RBC (0, 12.5, 25, 50, and 100%) mixed with IL at 37 °C. g) Storage modulus (G’) of the mixture of IL and various amounts of RBC (0, 12.5, 25, 50, and 100% v/v) (n = 3). h–j) Blood clotting index (BCI) of citrated whole blood (h), heparinized whole blood (i), and defibrinated whole blood (j) mixed with IL (−/+) or CaCl_2_ (−/+) (n = 6). Data are mean ± s.e.m. Statistical analysis was performed using one‐way ANOVA. ^**^ *p* < 0.01 and ^****^ *p* < 0.0001.

In this study, we introduce a novel liquid embolic material composed of polyethylene glycol (PEG) and a biocompatible ionic liquid (IL), named P‐LE. As illustrated in **Figure** **2**a, this innovative material is specifically designed to reach and effectively embolize the distal microvasculature, a challenging target often inaccessible with current clinical tools. P‐LE can achieve embolization in both normal as well as in anticoagulated blood (citrated, heparinized, or defibrinated), and this occlusion has a displacement force more than 7 times the normal systolic blood pressure. Our experiments using a rat femoral artery embolization model demonstrated P‐LE's ability to access distal microvasculature and maintain stable occlusion, even in anticoagulated blood. This finding suggests that thrombosis is not a prerequisite for successful embolization. These results were consistent in both terminal swine and anticoagulated canine embolization models, further underlining P‐LE's efficacy. P‐LE achieved embolization in blood vessels smaller than 4d microns, with the stability of the occlusion unaffected by anticoagulation. In a survival swine study, we evaluated the durability and safety of P‐LE, where it surpassed traditional clinically used microbeads in preventing recanalization and demonstrated a high safety profile. Further, in an acute porcine hemorrhage model, P‐LE rapidly achieved embolization, effectively obstructing blood flow in distal arterioles while preserving the integrity of upstream larger vessels.

## 2 Results

### 2.1 Characterization and Optimization of P‐LE

The study investigated a novel Ionic Liquid (IL) formulation composed of choline (C) and geranic acid (G) at varying molar ratios (2:1, 1:1, 1:2) for its potential as a small vessel embolic agent. This IL was analyzed for its interaction with blood and its ability to occlude blood vessels measuring 30–50 microns. Rheology experiments revealed that increasing geranic acid's ratio led to a significant rise in the gelation rate (G″), implying its role in enhancing coagulation or interaction with blood cells (Figure 2b). Further testing with separated blood components showed that plasma, white blood cells, and platelets had minimal impact on gelation (Figure 2c,d). However, mixing IL with the red blood cell (RBC) fraction caused immediate gelation, suggesting that RBCs are required for effective embolization (Figure 2e). The study also considered variable hematocrit levels in patients, which may result from factors including bleeding, chemotherapy, or radiotherapy. It was found that higher RBC levels corresponded with increased G″ levels, confirming the RBC dependency of the gelation process (Figure 2f,g). Additionally, the involvement of the coagulation cascade was examined using anticoagulated blood samples. The results indicated that the gelation of the blood‐IL mixture occurred independently of the coagulation cascade, as anticoagulated blood did not inhibit the gelation process (Figure 2h–j).

**Figure 3:**
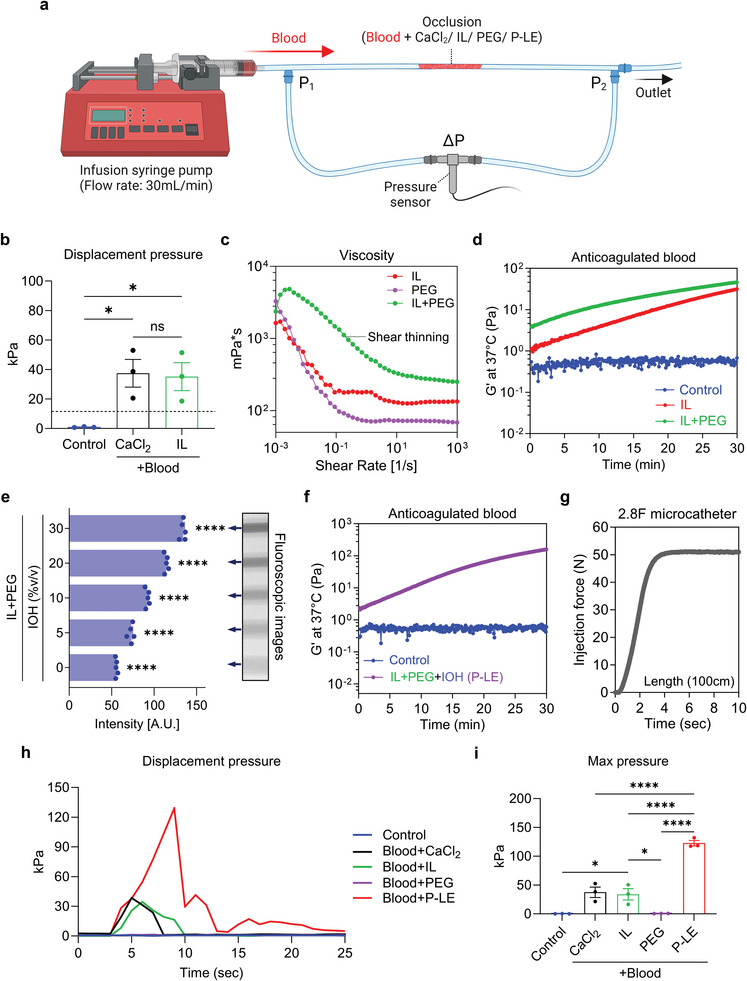
Optimization of P‐LE. a) The experimental setup for measuring the displacement pressures. The vessel occlusion was simulated inside a silicone tube and blood was infused at a flow rate of 30 mL min^−1^ with a syringe pump. b) The quantification of maximum pressure required to displace control (anticoagulated blood), a blood clot (anticoagulated blood+CaCl_2_), and anticoagulated blood mixed with IL (n = 3). c) Viscosity curves of IL, PEG, and IL+PEG characterized by shear rate sweeps. d) A hemorheology curves of anticoagulated blood mixed with PBS (control), IL, or IL+PEG at 37 °C. e) Fluoroscopic images of IL+PEG mixed with various concentrations of IOH (0, 5, 10, 20, and 30% v/v) and their corresponding intensities (n = 5). e) A hemorheology curve of anticoagulated blood mixed with P‐LE at 37 °C. g) An injection force of P‐LE to inject through a 2.8F microcatheter (length: 100 cm). h) Representative displacement pressure curves of various simulated vessel occlusions. i) The quantification of maximum pressure required to displace control (anticoagulated blood), a blood clot (anticoagulated blood+CaCl_2_), anticoagulated blood mixed with IL, PEG, or P‐LE (n = 3). Data are mean ± s.e.m. Statistical analysis was performed using one‐way ANOVA.^*^ *p* < 0.05, and ^****^ *p* < 0.0001.

The ideal embolic agent should only embolize the target site upon delivery from the catheter, avoiding migration or fragmentation and preventing unintended embolization. To assess the stability of the IL‐blood mixture, we conducted displacement pressure tests in a simulated blood flow system (**Figure** **3**a). These tests revealed that the IL‐blood mixture exhibited displacement forces comparable to blood clots formed by the addition of calcium chloride, indicating a potential risk of fragmentation or migration of the IL‐blood mixture (Figure 3b). To enhance the viscosity and stiffness of the IL‐blood gel, high molecular weight polyethylene glycol (PEG) was integrated into the IL formulation. Various amounts of PEG were dissolved in IL to determine the appropriate PEG levels to include in the final formulation. The viscosity measurement of IL with PEG showed the highest viscosity and the fastest blood gelation when the PEG concentration reached 100 mg mL^−1^. PEG concentrations higher than 100 mg mL^−1^ resulted in a precipitate, indicating that they exceeded the solubility threshold. This suggests that 100 mg mL^−1^ of PEG would be the optimal amount to include in the formulation. (Figure S1, Supporting Information). This addition significantly increased the viscosity of the IL, imparting a shear‐thinning property beneficial for catheter injection (Figure 3c). The IL‐PEG combination led to a quicker and more pronounced increase in modulus upon mixing with blood, compared to the IL alone (Figure 3d). Further investigation was conducted on the impact of this formulation on the activated clotting time (ACT) of anticoagulated blood, using different combinations of IL, PEG, and calcium chloride (CaCl_2_). The results showed that while CaCl_2_ induced thrombosis in anticoagulated citrated blood, lowering the ACT, PEG alone did not alter the ACT. However, any combination that included IL resulted in immediate thickening of the blood, underscoring IL's significant role in blood gelation, consistent with previous findings. These outcomes highlight IL's effectiveness as a key component in the formulation of the liquid embolic agent (Figure S2, Supporting Information).

To optimize the delivery of the IL+PEG mixture using fluoroscopic imaging in clinical settings, we incorporated iohexol (IOH), an FDA‐approved contrast agent, into the mixture. Fluoroscopic evaluation indicated that the ideal concentration of IOH was 30% by volume, resulting in a new formulation termed P‐LE (IL+PEG+30% IOH) (Figure 3e). Although the addition of 30% IOH slightly reduced the IL+PEG content in P‐LE, the formulation maintained its gelling properties (Figure 3f).

We also measured the injection force of P‐LE through a 2.8 F microcatheter, confirming its suitability for hand‐injection in endovascular procedures (Figure 3g). Further experiments focused on the speed of blood gelation with P‐LE, a crucial aspect for successful vessel occlusion in medical applications. Tests with anticoagulated citrated blood demonstrated that P‐LE induced instant gelling of the blood in comparison to the control and embolization coil group, indicating that P‐LE is a potent embolic agent capable of functioning effectively even in patients with coagulopathic conditions, an outcome that holds considerable significance in clinical settings. Furthermore, when P‐LE is injected within the coil mass of a failed coil embolization using a microcatheter, the combination results in the immediate gelation of blood. This finding is particularly significant in clinical scenarios where coil embolization fails in coagulopathic patients; however, with P‐LE, these failed cases can be rescued (Figure S3, Supporting Information).

### 2.2 Ex Vivo P‐LE Displacement Test

To evaluate the stability of a P‐LE‐mediated vascular occlusion against the forces exerted by systolic blood pressure, displacement pressure measurements were conducted. These measurements were consistent with the rheology findings shown in Figure 3d. When blood was combined with IL or calcium chloride, the resulting blood clots exhibited displacement forces that indicated a potential risk for fragmentation. In contrast, blood mixed with PEG as part of the P‐LE formulation exhibited significantly greater resistance. The measured displacement forces, in this case, were eight times higher than the normal systolic blood pressure, suggesting robust stability of the embolization in vivo (Figure 3h,i).

### 2.3 P‐LE Embolization in Heparinized Rat Femoral Artery

**Figure 4:**
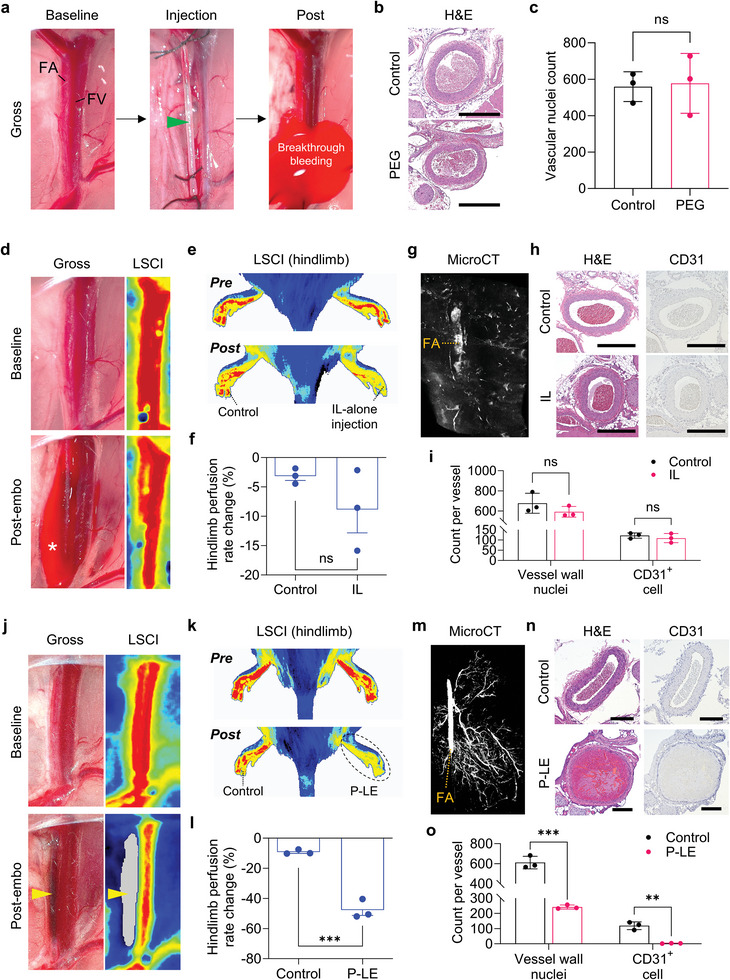
PEG, IL, or P‐LE injection in rat femoral artery embolization model. a) Rat femoral artery embolization performed with PEG only. Severe breakthrough bleeding from the needle‐puncture hole was observed post‐injection, suggesting that the PEG does not obstruct blood flow when used alone. b) Representative H&E images of untreated and PEG‐injected FA. PEG‐injected arteries appear to be normal with no sign of occlusion. c) The quantification of vascular nuclei demonstrates no significance between control and PEG groups (n = 3). d) Rat FA embolization performed with IL alone. Minor breakthrough bleeding from the needle‐puncture hole was observed post‐injection of IL, suggesting that IL‐alone injection resulted in an incomplete occlusion of FA (the asterisk shows the breakthrough bleeding). Laser speckle contrast imaging (LSCI) showed minimal perfusion change in FA pre‐ and post‐embolization. e) Representative hindlimb LSCI images showing minimal changes of perfusion after embolization with IL. f) The quantification of hindlimb perfusion rate in control and embolized hindlimb before and after IL injection (n = 3). g) Micro‐CT image showing poor embolization in FA and its downstream microvessels; the majority of IOH has been washed out. h) Representative H&E and CD31 immunostaining images of control and IL‐injected FA. i) The quantitative analysis of vascular nuclei and CD31‐positive cells of IL‐injected FA (n = 3). j) Gross and LSCI analyses of rat FA embolization showing the persistent embolization using P‐LE (yellow arrows indicate the created embolus). k) LSCI images of control and embolized rat hindlimbs pre‐ and post‐embolization with P‐LE. l) Hindlimb perfusion rate change of control and embolized limbs compared to respective baseline measurements (n = 3). m) Micro‐CT image showing the embolization of rat FA and its downstream microvessels with P‐LE. n) Representative H&E and CD31 immunostaining images of untreated and embolized FA with P‐LE. o) Average count of vessel wall nuclei and CD31^+^ cells per vessel for control and embolized FA (n = 3). Scale bars: (b) and (h), 500 µm. n) 250 µm. The images shown are representative of the 3 mice analyzed per group. Data are mean ± s.e.m. Statistical analysis was performed using an unpaired *t*‐test in c, f, and l, and multiple unpaired *t*‐tests in i and o. ns, not significant; ^**^ *p* < 0.01 and ^***^ *p* < 0.001.

The durability and effectiveness of P‐LE‐mediated embolization in rat femoral arteries were investigated, focusing on the extent of distal small vessel embolization (Figure S4, Supporting Information). Additionally, to demonstrate that P‐LE embolization is independent of coagulation, experiments were conducted using heparinized rats. For control purposes, FA embolization with either PEG or IL alone was initially performed. Post‐PEG injection into the FA, there was notable breakthrough bleeding from the puncture site, indicating the absence of vessel occlusion (**Figure** **4**a). On histology, the FA was patent with no embolization and vessel wall ablation, supporting our in vitro findings that PEG alone is ineffective as an embolic agent (Figure 4b,c). Similarly, FA embolization with IL alone also resulted in breakthrough bleeding, suggesting incomplete embolization. Laser Speckle Contrast Imaging (LSCI) revealed minimal changes in arterial blood flow following IL injection, compared to baseline levels (Figure 4d). Hindlimb perfusion assessments indicated an insignificant change in perfusion rate after IL‐only embolization, implying insufficient embolization (Figure 4e,f). High‐resolution Micro‐CT imaging demonstrated that embolization with IL alone was not sustained, as it was predominantly washed out from the FA and its side branch microvessels (Figure 4g). Histologically, the IL‐injected FA also remained patent with vessel wall cellularity similar to the untreated control and to the PEG injection. CD31 immunostaining showed no significant difference between the control and IL‐injected FA, also indicating the lack of vessel occlusion (Figure 4h,i). Collectively, these findings suggest that neither PEG nor IL alone can achieve complete embolization.

When PEG and IL are combined in the P‐LE formulation, embolization was successful without any breakthrough bleeding (Figure 4j). This was confirmed by hindlimb LSCI analysis demonstrating reduced blood perfusion in the embolized limb, suggesting on‐target embolization was achieved by P‐LE (Figure 4k,l). Following embolization, the FA and its downstream branches were imaged using micro‐CT to visualize whether P‐LE was able to reach distal microvessels. Analysis of micro‐CT images showed that P‐LE can reach microvessels smaller than 40 microns in diameter (Figure 4m). In contrast, Obsidio gel embolic was not able to reach sub‐mm downstream vessels and mostly remained upstream (Figure S5, Supporting Information). On H&E and endothelial cell (CD31) immunostaining images, P‐LE‐embolized FA showed significantly reduced vascular cellularity and CD31 expression, indicating the complete and rapid embolization of FA and vessel wall ablation using P‐LE (Figure 4n,o). Next, FA embolization was repeated in heparinized (250U/kg) rats to evaluate P‐LE embolization in an anticoagulated state. In heparinized rats with significantly increased activated clotting time (ACT) (Figure S6a, Supporting Information), embolization was consistently successful indicating that coagulation is not necessary for P‐LE embolization to occur (Figure S6b–g, Supporting Information). These findings suggest that the P‐LE formulation can reach the microvasculature when the embolic material is delivered in upstream larger blood vessels and successfully embolizes them.

### 2.4 P‐LE in Porcine Renal Embolization Model

**Figure 5:**
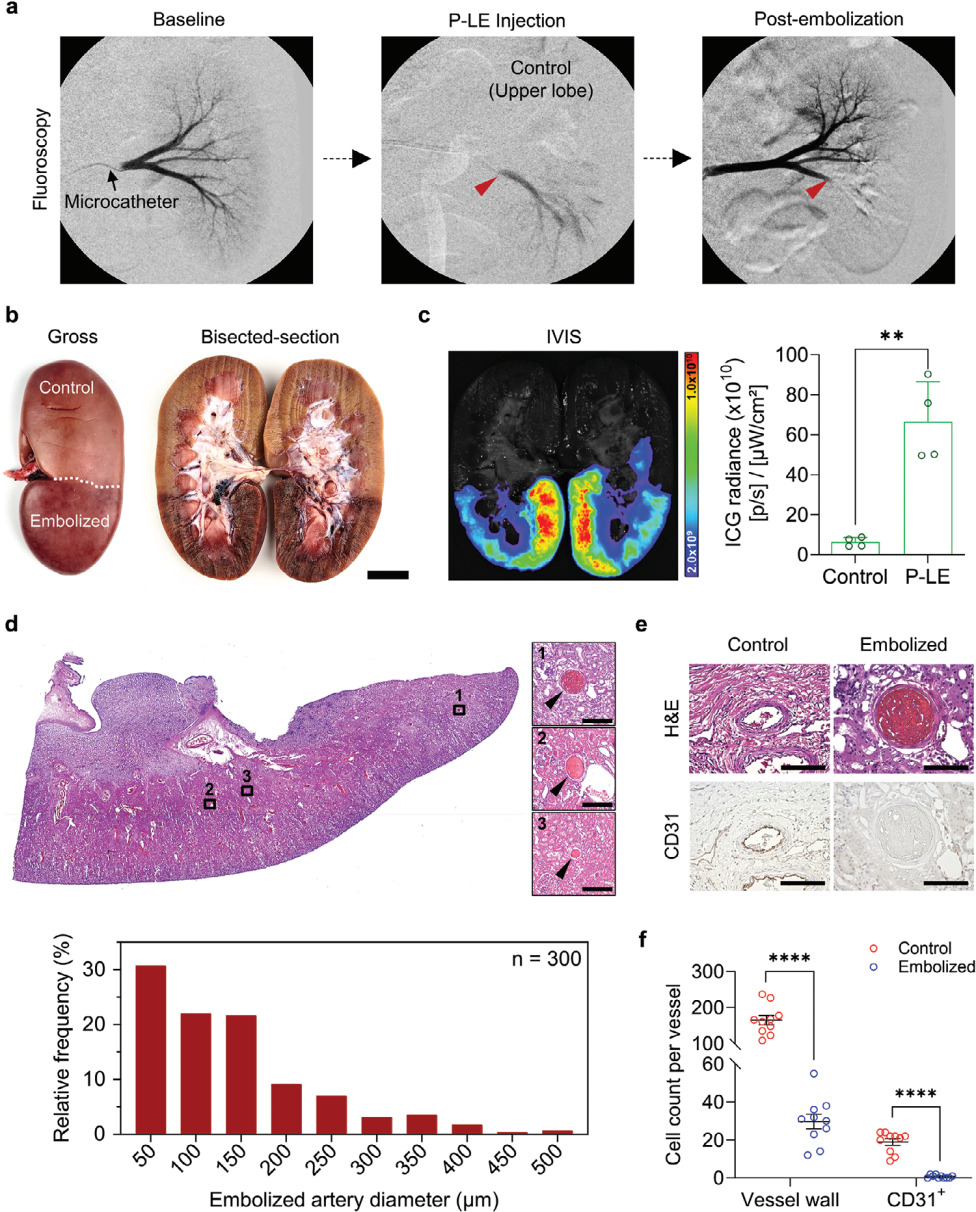
P‐LE injection in porcine renal embolization non‐survival model. a) Representative DSA images showing arterial anatomy of a porcine kidney before injecting P‐LE (baseline), during the injection of P‐LE to the lower lobe of the kidney, and post‐embolization (arrows indicate the site of P‐LE injection). b) Gross images of whole and bisected kidneys showing discoloration in the lower lobe embolized with P‐LE demarcating the area of injection. c) IVIS image of a bisected kidney showing ICG signals localized in the lower renal cortex region, and the quantification of ICG radiance in control (non‐treated lobes) and P‐LE‐embolized lobes (n = 4). d) H&E image of renal cortex embolized with P‐LE and its representative magnified images showing embolized microvessels (top). Relative frequencies of embolized vessels in various diameters were quantified (n = 300). e) Representative H&E and CD31 immunostaining images of vessels in control and embolized kidneys. f) The count of total cells in vessel walls and CD31^+^ cells per vessel in control and embolized kidneys (n = 10). Scale bars: (b), 3 cm. d) 200 µm. e) 100 µm. The images shown are representative of the 4 pigs analyzed per group. Data are mean ± s.e.m. Statistical analysis was performed using an unpaired *t*‐test in c, and multiple unpaired *t*‐tests in f. ^**^ *p* < 0.01 and ^****^ *p* < 0.0001.

To determine whether P‐LE can reach the distal vasculature and embolize them, a porcine kidney embolization model was used because of its characteristic hierarchical vascular network. Under real‐time fluoroscopic guidance, a microcatheter was navigated to the proximal portion of the main lower pole renal artery and ≈2 cc of the P‐LE was injected over 30 seconds (**Figure** **5**a; Video S1, Supporting Information). During injection of P‐LE, Figure 5a demonstrates clear visibility of the embolic agent and opacification of the distal parenchymal vessels in the cortex suggesting that it is reaching the intended smaller vessels. Right after P‐LE delivery, digital subtraction angiography (DSA) was performed, showing the absence of flow to the lower pole of the kidney, indicating instant and complete embolization. At necropsy, a gross examination of the kidney revealed a geographic demarcation of the embolized lower pole of the kidney; this was more obvious in the bisected kidney showing the dark discoloration of the embolized regions of the lower kidney segment (Figure 5b). P‐LE embolized renal arteries were also distinctively visible on gross inspection (Figure S7, Supporting Information). Detection of indocyanine green (ICG) fluorescence overlapped with the discolored portions of the kidney confirming that the ICG signal was delivered to the lower pole kidney via P‐LE (Figure 5c). On histology, more than 50% of the embolized vessels measured up to 100 microns in diameter with 30% 50 microns or less suggesting that P‐LE is an effective embolic agent of microvessels (Figure 5d). CD31 staining showed normal endothelium cellularity in the untreated controls, while vessels embolized with P‐LE demonstrated the absence of CD31 detection with significant vessel wall ablation (Figure 5e,f). As an indirect measure of successful embolization, immunostaining for hypoxia‐inducible factor‐2α (HIF‐2α) was performed suggesting detection of hypoxia surrounding embolized blood vessels (Figure S8, Supporting Information). To further show that P‐LE can reach distal microvessels and embolize them, P‐LE was injected into the splenic artery; DSA images in Figure S9 (Supporting Information) clearly showed that P‐LE successfully embolized the splenic sinusoids while maintaining flow in the main branch splenic artery.

### 2.5 P‐LE in Anti‐Coagulated Canine Renal Embolization Model

**Figure 6:**
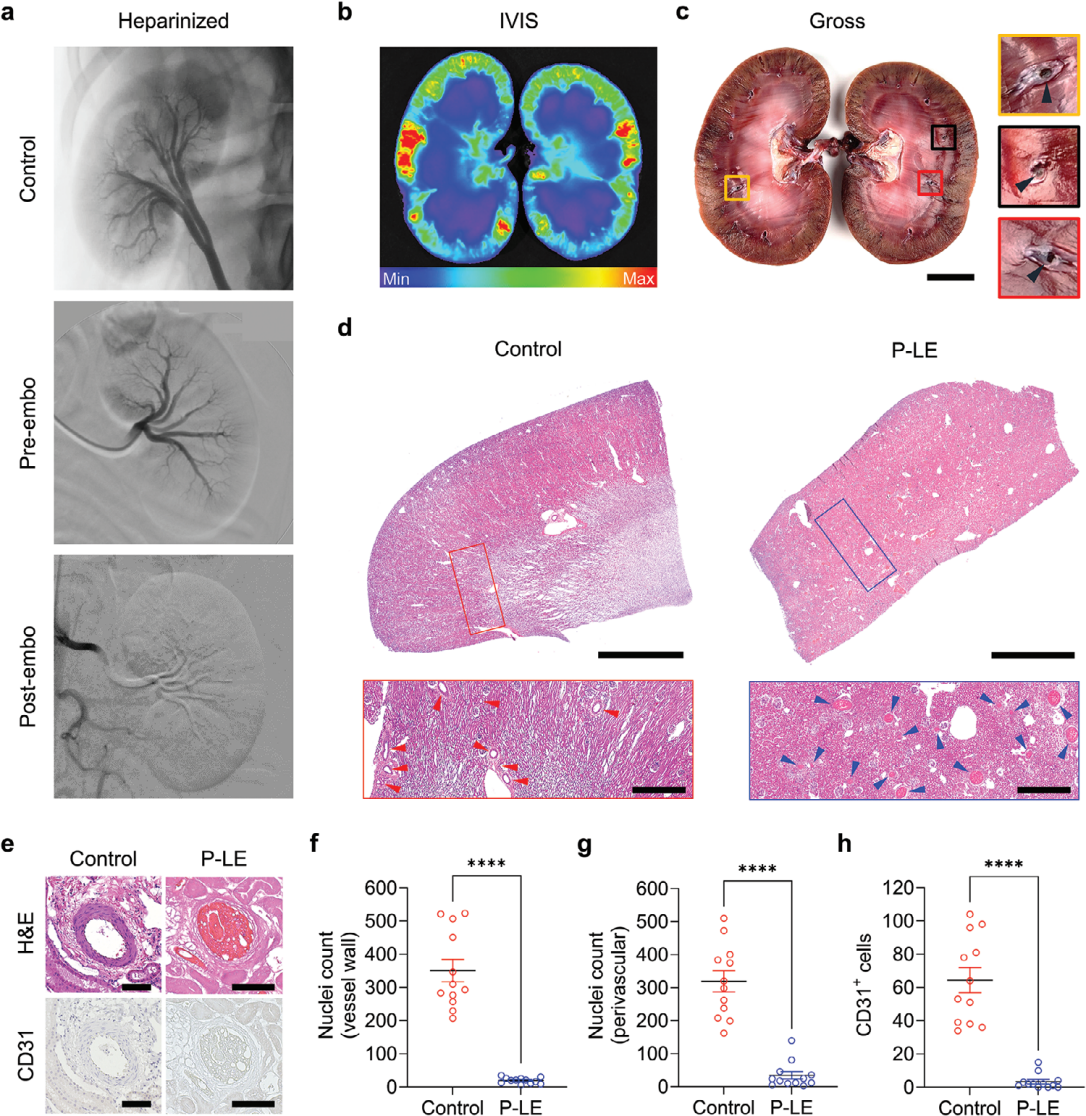
P‐LE injection in heparinized canine renal embolization non‐survival model. a) Representative DSA images showing arterial anatomy of a heparinized canine with no embolization (control) and pre‐ and post‐embolization with P‐LE. b) IVIS image of a bisected kidney showing ICG fluorescence from P‐LE formulation localized in the renal parenchyma after embolization. c) Gross image of a bisected kidney and its representative magnified images (yellow, black, and red boxes) showing the embolized vessels (indicated by black arrowheads). d) H&E images of whole tissue sections and their magnified images (red and blue boxes) of kidney parenchyma in control and embolized kidneys. Arrowheads indicate microvessels in control (red) and embolized (blue) kidneys, respectively. e) Representative H&E and CD31 immunostaining images of vessels in control and P‐LE‐injected kidneys. f–h) Average nuclei count in vessel walls (f) and perivascular regions (g), and CD31^+^ cells per vessel in non‐embolized (control) and embolized kidneys (n = 12). Scale bars: (c), 2 cm. d) 6 mm (top) and 1 mm (bottom). e) 100 µm. The images shown are representative of the 4 canines analyzed per group. Data are mean ± s.e.m. Statistical analysis was performed using an unpaired *t*‐test in (f–h). ^****^ *p* < 0.0001.

To simulate the clinical scenario of anticoagulation or coagulopathy, canines received IV heparin (250U/kg) resulting in a significantly increased ACT (Figure S10, Supporting Information). Using a combination of a guide catheter and a microcatheter, P‐LE was slowly injected from the main renal artery. Post‐embolization angiography demonstrated successful embolization of the entire kidney using P‐LE (**Figure** **6**a). On IVIS imaging, ICG distribution was seen across the parenchyma of the renal cortex, suggesting that the P‐LE likely reached the distal target microvessels (Figure 6b). Gross evaluation of the bisected kidneys clearly demonstrated embolized vessels (Figure 6c) and on histology, uniform embolization of sub‐mm microvessels as small as 50 microns in diameter were noted, consistent with the porcine model of embolization (Figure 6d). Representative H&E images and immunostaining for CD31 showed that while untreated control vessels had normal cellularity and CD31 expression, the arteries that received P‐LE showed absence of CD31 detection and reduced cellularity suggesting vessel wall ablation (Figure 6e–h).

### 2.6 P‐LE in Porcine Survival Renal Embolization Model

**Figure 7:**
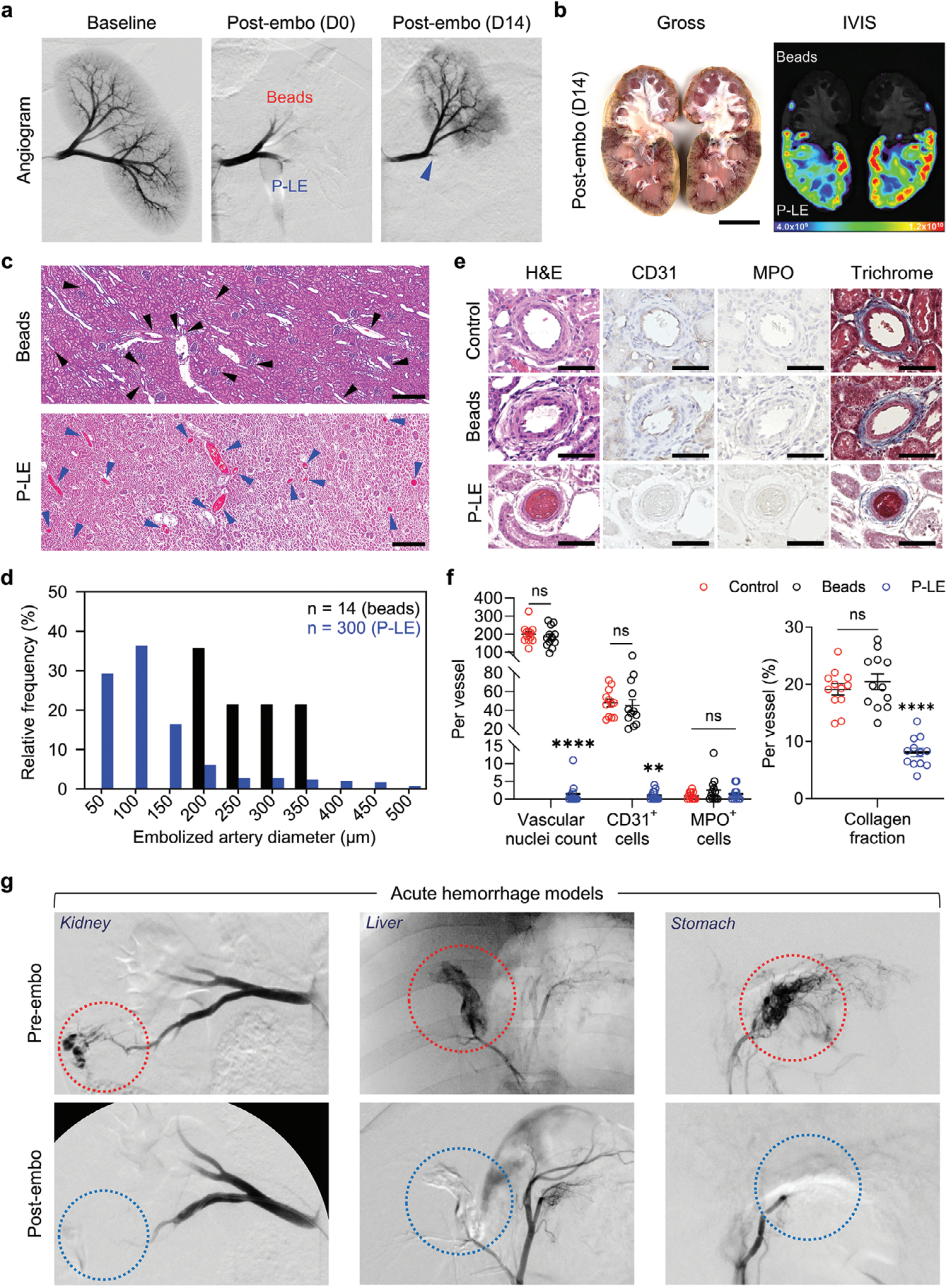
P‐LE injection in porcine renal embolization survival model. a) Baseline and post‐embolization (D0 and D14) DSA of a porcine kidney. The upper pole of the kidney was injected with clinically used 300 micron microbeads (Embosphere) and the lower pole was injected with P‐LE. Both showed successful initial embolization (D0), but the upper lobe injected with microbeads was recanalized at D14 while P‐LE showed persistent embolization. b) Gross and IVIS images of a bisected kidney showing discoloration and ICG localization in the lower pole of the kidney demonstrating permanent embolization by P‐LE. c) Representative H&E images of renal cortex parenchyma in beads and P‐LE‐injected kidneys. Arrowheads indicate microvessels in beads (black) and P‐LE‐injected (blue) kidneys, respectively. d) Relative frequencies of embolized microvessels. Only 14 vessels were shown to remain occluded with beads indicating that the majority was patent. e) Representative H&E, CD31, and MPO immunostaining and trichrome images of microvessels suggesting persistent embolization of microvessels with P‐LE. f) Quantitative analysis of vascular nuclei count and CD31 immunostaining from microvessels show significant differences with P‐LE embolization. Quantification of MPO immunostaining shows no significance among all tested groups. Quantitative analysis of collagen fraction in microvessels demonstrated significantly lower collagen content in P‐LE‐embolized vessels indicating persistent occlusion of vessels (n = 12). g) Fluoroscopic images of hemorrhaging microvessels and post‐embolization with P‐LE. Immediate hemostasis was achieved with P‐LE embolization. Scale bars: (b), 3 cm. c) 500 µm. e) 50 µm. The images shown are representative of the 6 pigs analyzed per group. Data are mean ± s.e.m. Statistical analysis was performed using two‐way ANOVA in (f) (left) and one‐way ANOVA in f (right). ns, not significant; ^**^ *p* < 0.01 and ^****^ *p* < 0.0001.

Porcine survival experiments were performed to evaluate the durability and superiority of P‐LE embolization compared to the clinically used microsphere embolization (Embosphere). Beads were suspended in a solution with equal amounts of IOH and ICG and then injected to stasis into the upper pole of the kidney; the lower pole of the same kidney received P‐LE (**Figure** **7**a). Subsequent DSA imaging demonstrated successful embolization. DSA imaging at day 14 revealed recanalization of the upper pole renal artery that received Embospheres, consistent with the clinical experience. In contrast, the lower pole that received P‐LE remained embolized at day 14 suggesting permanent embolization with no recanalization (Figure 7a; Video S2, Supporting Information). At necropsy, discoloration of the P‐LE embolized tissue overlapped with ICG detection suggesting persistent delivery of the ICG to the renal cortex (Figure 7b; Figure S11, Supporting Information). On histology, the renal cortex that received Embospheres showed mostly viable tissue with patent microvessels, while tissue that received P‐LE demonstrated uniform embolization with >60% of arteries up to 100 microns containing intra‐luminal P‐LE and ablation of the parenchyma tissue (Figure 7c–e). On histology and immunostaining for CD31, vessels embolized with P‐LE demonstrated significantly reduced vascular cellularity and absence of CD31 detection. No notable difference was observed with MPO immunostaining in the perivascular space; however, significant MPO staining was observed in the renal capsule, which is consistent with the renal capsular region having its own independent blood supply (Figure 7e,f; Figure S12, Supporting Information). Trichome images of microvessels and their quantitative analyses showed significantly lower collagen content in P‐LE‐embolized vessels further confirming the permanent obstruction of blood flow (Figure 7e,f).^[^ ^10^ ^]^ P‐LE demonstrated no toxicity up to day 14 compared to baseline and immediately post‐embolization. Oxygen saturation (SpO_2_), ACT, complete blood cell (CBC) counts, and serum biochemical analysis showed no unusual changes suggesting biocompatibility of P‐LE (Figure S13, Supporting Information).

### 2.7 Evaluation of P‐LE in Acute Porcine Hemorrhage Model

To determine the efficacy of P‐LE in embolizing acutely bleeding microvessels, porcine acute microvascular hemorrhage models were created in the kidney, liver, and stomach. Once the bleeding was confirmed by contrast extravasation, the microcatheter was selectively navigated to the bleeding site, and P‐LE was injected to achieve instant embolization and treatment of the hemorrhage. Post‐embolization fluoroscopic imaging demonstrated that the embolization was successful with rapid hemostasis (Figure 7g). These experiments indicate that in an acute scenario, P‐LE can successfully treat hemorrhage.

### 2.8 P‐LE Susceptibility Tests to Drug‐Resistant Pathogens

In clinical practice, abscess formation is an uncommon but potentially fatal long‐term complication of embolization procedures.^[^ ^11^ ^]^ To determine whether P‐LE can prevent potential post‐embolization related infections, susceptibility tests were performed using some of the most antibiotic‐resistant bacteria isolated from patients at the Mayo Clinic. P‐LE demonstrated significant antimicrobial effects on all drug‐resistant superbugs tested including, methicillin‐resistant *Staphylococcus aureus* (MRSA; minimum inhibitory concentration (MIC), 0.097%), vancomycin‐resistant *Enterococcus faecium* (VRE; MIC, 0.39%), and carbapenem‐resistant *Enterobacterales* (CRE; MIC, 0.39%), *Clostridioides* *difficile* (*C. difficile*; MIC, 0.39%), and *Candida auris* (*C. auris*; MIC, <0.049%) (Figure S14, Supporting Information). These exceptional antimicrobial effects of P‐LE suggest that while conventional embolics may be at risk of procedural infections, P‐LE may protect patients from potential septic complications.

## 3 Discussion

Microvascular hemorrhage is a common cause of clinical bleeding that is challenging to treat using embolics in clinical practice today. Given the widespread use of anticoagulants, the prevalence of microvessel bleeding is continuing to increase. For example, spontaneous bleeding from the psoas muscle, liver, or splenic bleeding from minor trauma or bleeding from tumors are common at tertiary medical centers and they represent some of the most challenging vascular beds to embolize. In this context, we explored the effectiveness of a bioengineered formulation, P‐LE, in embolizing microvessels as small as 40 microns in tissue parenchyma, even under anti‐coagulated conditions. We hypothesized, consistent with our experiments, that P‐LE induces the aggregation of local proteins and gelation of the blood, primarily through interactions with red blood cells. When P‐LE is injected into the bloodstream, it likely interacts with red blood cells, causing the release of large amounts of red cell‐derived proteins such as hemoglobin and membrane proteins.^[^ ^12^ ^]^ This interaction may accelerate the embolization process, offering a potential solution for effectively managing microvascular hemorrhages. To enhance the viscosity of the injected liquid embolic and reinforce the vessel occlusion to withstand systolic pressure reliably, we integrated high molecular weight PEG. This modification significantly increased the viscosity of the embolic agent, while also imparting a shear‐thinning quality, allowing for smooth injection through a microcatheter. P‐LE was ≈ 8 times more resistant to displacement indicating that fragmentation and migration will likely not occur, which was also shown to be the case in vivo. For better visualization during procedures, we included a clinically approved contrast agent, IOH, in P‐LE's formulation. We also tested P‐LE's effectiveness in various animal models, including rats, swine, and canines, both in normal and heparinized conditions. These tests demonstrated P‐LE's rapid and effective embolization of microvessels. In a survival study using a swine renal embolization model, P‐LE outperformed clinically used microbeads in blocking vessels measuring 50 microns, with no signs of recanalization, displacement, fragmentation, or migration. Additionally, biocompatibility studies conducted over 14 days showed no significant toxicity from P‐LE injections. P‐LE's potential was further highlighted in acute hemorrhage models in porcine kidneys, livers, and stomachs, where it rapidly achieved hemostasis. This suggests its usefulness in treating microvascular hemorrhage, even under anticoagulation conditions, suggesting that it could potentially save patients in critical situations. Furthermore, P‐LE exhibited effectiveness against various strains of patient‐derived drug‐resistant pathogens at concentrations as low as 0.049%, indicating its potential in preventing septic complications. This was expected, as P‐LE is primarily composed of IL. Recent studies have shown that ILs exert a potent antimicrobial effect by disrupting bacterial cell membranes through electrostatic interactions.^[^ ^13^ ^]^ This multifaceted approach of P‐LE addresses the immediate need for effective embolization and offers broader implications for patient safety and post‐procedural care.

There were several limitations to our study. Microvessel hemorrhage is often seen in chronic infectious and neoplastic conditions, as well as in minor trauma in patients receiving anticoagulation; our model of hemorrhage, however, included direct trauma to the parenchyma where small vessels reside. While our experiments aimed to show that P‐LE could travel to where these small vessels are located, we could not test them in similar clinical scenarios in our large animal models. Additionally, our survival experiments following P‐LE embolization lasted 14 days; while 2 weeks is a good measure of durability, future experiments should include longer survival experiments of up to 6 months to measure recanalization and potential systemic toxicity.

## 4 Experimental Section

### Preparation and Optimization of P‐LE

The ionic liquid was prepared by a metathesis reaction of geranic acid (Sigma–Aldrich, St. Louis, MO) and choline bicarbonate (Sigma‐Aldrich). Initially, neat geranic acid was purified using the recrystallization method at a temperature of −80 °C in acetone. Subsequently, the purified geranic acid was combined with choline bicarbonate at predefined ratios of 2:1, 1:1, or 1:2. The mixture was stirred at 25 °C until the handheld carbon dioxide probe (GM70, VAISALA, Vantaa, Finland) indicated the absence of CO_2_ byproduct. Following the completion of the reaction, any remaining H_2_O was removed using a rotary evaporator (R‐300, Buchi, New Castle, DE) at 60 °C for 1 h. To enhance IL's viscosity and promote blood gelation, 100 kDa polyethylene glycol (PEG, Sigma‐Aldrich) was added (100 mg mL^−1^). To completely dissolve PEG in IL, neat PEG was added to IL and magnetically stirred at 60 °C for 48 h. Anti‐coagulated blood (Innovative Research Inc., Novi, MI) was mixed with each IL or IL+PEG mixture (1:1 v/v). The hemorheology of mixture samples was measured using a rheometer (MCR 302, Anton Paar, Torrance, CA) at 37 °C. A rheometer was outfitted with a sandblasted 25‐mm aluminum shaft (Anton Paar) and aluminum plate (Anton Paar), maintaining a 1 mm gap in between. Storage modulus measurements were conducted on all samples for 30 min. Then, different amount of the FDA‐approved contrast agent Omnipaque (IOH, GE Healthcare Systems, Chicago, IL) was incorporated, and the samples were subjected to fluoroscopy (OEC Elite C‐Arm, GE Healthcare Systems) for real‐time visualization during the embolization procedure. The intensity of the fluoroscopic signal of different concentrations of IOH was quantified using ImageJ (National Institutes of Health, Bethesda, MD). Upon finalizing the formulation of P‐LE, hemorheology measurement was repeated to ensure that the addition of IOH did not hinder the ability of liquid embolic to gelate blood. For in vivo studies, ICG (United States Pharmacopeia, North Bethesda, MD) was incorporated into P‐LE at a final concentration of 0.25 mg mL^−1^ to assess P‐LE delivery post‐embolization.

### Blood Gelation Assay using P‐LE and Clinically used Coils

Saline (Baxter Healthcare Corporation, Deerfield, IL), or P‐LE was mixed with anti‐coagulated blood (1:1 v/v) with or without an embolic coil (Tornado embolization microcoil, Cook Medical, Bloomington, IN) in a 12‐well plate. The samples were incubated at 37 °C and at predetermined time points, all wells were washed 3 times using saline to remove residues before imaging.

### Injection Force Measurements

The injectability of P‐LE was determined using a mechanical tester (Instron 5942, Instron, Norwood, MA). P‐LE was loaded in 1 cc syringe (Medallion, Merit Medical, South Jordan, UT) and injected through a 100 cm 2.8 F microcatheter (PROGREAT, Terumo Interventional Systems, Somerset, NJ) at a constant flow rate of 1 mL min^−1^. The injection force was recorded using the Bluehill version 3 software (Instron).

### Displacement Pressure Measurements

The displacement pressure of the created *ex vivo* embolus was measured with a custom‐built displacement pump set‐up (WPI; pressure sensor from PASCO Capstone). Tested materials (IL, PEG, and P‐LE) were mixed with blood, and 1cc of the mixture was quickly placed at the center of the silicone tubing using a 2.8F microcatheter. After assembling the set‐up, the infusion syringe pump (Kent Scientific, Torrington, CT) loaded with a 50 mL syringe (Henke Sass Wolf, Tuttlingen, Germany) filled with anti‐coagulated blood was activated at a constant 30 mL min^−1^ flow rate. All measurements were performed 3 times for quantitative analysis.

### Blood Clotting Index (BCI) Assessments

Anticoagulated blood was first placed in a 10 mL glass vial (Duran Wheaton Kimble, Rockwood, TN) and P‐LE was slowly added (10% v v^−1^) with or without the addition of calcium chloride (CaCl_2_, 30:1, v/v, Sigma–Aldrich). After gentle mixing, the samples were incubated at 37 °C for 5 min. Saline was gently added to the samples without disrupting the formed clots. BCI was calculated as, BCI = 100%‐(A_450_ of the sample's supernatant/ A_450_ of negative control) x 100%. Each measurement was repeated at least 5 times using citrated, heparinized (Innovative Research Inc.), and defibrinated (Innovative Research Inc.) blood for quantitative analysis.

### Activated Clotting Time (ACT) Assessments using different Blood Components

EDTA‐blood (Innovative Research Inc.) was used to obtain each blood component. Platelet‐poor plasma was prepared by centrifugation at 3000 g for 10 min. Platelet‐rich plasma (supernatant) and red blood cells (cell pellets) were prepared by centrifugation at 2,000 rpm for 10 min. Prepared separate blood components were mixed with P‐LE (10% v v^−1^) with or without CaCl_2_ and immediately loaded into the Celite ACT cartridge (Abbott Laboratories, Chicago, IL) and the i‐STAT handheld blood analyzer (Abbott Laboratories) for the measurements.

### Femoral Artery Embolization in Normal and Heparinized Rat Model

All animal experiments were conducted in accordance with the animal protocols approved by the Mayo Clinic Institutional Animal Care and Use Committee (IACUC; approval: A00006995‐23). Sprague‐Dawley rats with weights ranging from 300 to 400 g were randomly divided for the femoral artery embolization procedure. The animals were anesthetized via inhalation of 2 L min^−1^ of isoflurane (Piramal Critical Care, Mumbai, India) while resting on a warming pad. Positioned in a supine posture, the hair in the inguinal region was clipped and applied with povidone–iodine (Purdue Products, Stamford, CT). A 2.0 cm incision was created in parallel with the inguinal ligament to expose the femoral artery. A vascular sheet was removed, and the proximal FA was ligated using a 7‐0 silk suture (Teleflex Medical, Plymouth, MN). Two ligatures were positioned distally on the FA for the injection of the embolic material to be tested. Within the space delineated by the two distal ligatures, a 30‐gauge syringe was used for the intra‐arterial injection of either PEG, IL, or P‐LE. Following the injection, the proximal ligature was promptly released to allow blood flow into the injected materials. 5 min after the removal of the proximal FA ligature, the distal ligatures were also removed to evaluate blood vessel occlusion by monitoring for any signs of breakthrough bleeding through the needle puncture site. For heparinized rats, a dosage of 250U/kg of heparin (Fresenius Kabi, Lake Zurich, IL) was administered 10 min prior to performing the embolization procedure. Hindlimb and FA of all rats were subjected to gross examination and laser speckle contrast imaging (LSCI, PeriScan PIM 3, Perimed Inc., Las Vegas, NV). At necropsy, the FA and surrounding tissues were harvested and examined under a micro‐CT scanner (Skyscan 1276, Bruker, Kontich, Belgium) to visualize the distribution of injected materials.

### Renal Embolization in Non‐Survival and Survival Porcine Model

Renal artery embolization was performed as previously described.^[^ ^14^ ^]^ (approval: A00002532‐17‐R22). Briefly, Yorkshire pigs (Premier BioSource, Ramona, CA) with a weight range of 50–60 kg were acclimated for 7 days under the supervision of a veterinarian. Before the procedure, pig anesthesia was initially induced through intramuscular injection of 5 mg kg^−1^ tiletamine‐zolazepam (Telazol, Zoetis, Parsippany‐Troy Hills, NJ), 2 mg mL^−1^ xylazine, and 0.02 mg kg^−1^ glycopyrrolate, and then maintained with 2% of isoflurane inhalation. To access the renal artery, the iliac artery was initially accessed under the guidance of ultrasound imaging (Butterfly iQ+, Butterfly Network Inc., Guilford, CT). Subsequently, a guidewire (Cook Medical) was carefully placed. Real‐time digital subtraction angiography (DSA) was used to guide and accurately position a 2.8 F microcatheter (Terumo Interventional Systems, Somerset, NJ) within the targeted lower renal artery branch. P‐LE was introduced into the lower lobe of the kidney under real‐time fluoroscopic imaging. For survival study, clinically used 300 micron Embosphere was injected into the upper renal lobe for comparison. After the P‐LE injection, DSA was repeated to confirm the successful embolization of targeted renal artery branches. The pigs were humanely euthanized with Euthasol (Virbac, Westlake, TX) 1 h after embolization for the nonsurvival study and 14 days after embolization for the survival study, respectively. During necropsy, all kidneys were harvested and bisected for gross examination, as well as for IVIS spectrum in vivo imaging system (PerkinElmer Inc., Waltham, MA) imaging. The IVIS imaging was taken using an ICG filter (740/850 nm) and quantified using Living Image software (PerkinElmer).

### Renal Embolization in a Non‐Survival Heparinized Canine Model

Mongrel breed canines (Tri‐Valley Resources, LLC, Spring Green, WI) with weights ranging from 35 to 50 kg were randomly divided for the embolization procedure (approval: A00007058‐23). To sedate animals, an intramuscular injection of a cocktail comprising 0.2 mg kg^−1^ Midazolam (Avet Pharmaceutical, East Brunswick, NJ), 0.1 mg kg^−1^ Butorphanol (VCA Inc., Los Angeles, CA), and 5 mg kg^−1^ Propofol (Zoetic Inc., Richmond VA) was used. To induce heparinization in the canines, a dose of 250U kg^−1^ of heparin was administered 10 min before the procedure. The procedure for accessing the renal artery of the canines was performed identically to porcine models. Given the unique kidney structure in canines, the entire kidney was subjected to embolization. The canines were humanely euthanized 1 h after the embolization, and all kidneys were collected for gross examination and IVIS imaging.

### Histological and Immunohistochemical (IHC) Analysis

The tissues were fixed in 10% buffered formalin (Fisher Scientific, Waltham, MA) for a minimum of 7 days before being prepared for paraffin embedding. Tissue sections were sliced to a thickness of 5 µm and underwent a hydration process by sequentially immersing them in xylene (Fisher Scientific), followed by a series of ethanol (Fisher Scientific) solutions at concentrations of 100%, 95%, 80, 70, and 50%, and finally distilled water. For immunostaining of the slides, previously hydrated slides were placed into a 10 mm sodium citrate buffer and subjected to boiling at 95 °C for 30 min followed by 30 min of cooling down for antigen retrieval. The slides were washed with 3 changes of distilled water and background quenching was performed by immersing them in a solution of 3% hydrogen peroxide (Sigma‐Aldrich) in 60% methanol (Fisher Scientific) for 30 min at room temperature. Subsequently, the slides were washed with 0.05% PBS‐T for 5 min and then blocked for 1 h using 5% goat serum (50197Z, Thermo Fisher Scientific). After the blocking step, CD31 (ab182981, 1:1000, Abcam), HIF‐2α (ab109616, 1:1000, Abcam), MPO (ab208670, 1:500, Abcam) primary antibodies in 5% goat serum were pipetted onto each slide and incubated overnight at 4 °C. On the following day, the slides were consecutively washed three times with 0.05% PBS‐T. Horseradish peroxidase (HRP)‐conjugated goat anti‐rabbit IgG H&L (ab97051, Abcam) at a 1:500 dilution was then applied to the tissue sections and incubated at room temperature for 1 h. Afterward, the samples were once again washed three times with 0.05% PBS‐T. Subsequent 3,3′‐Diaminobenzidine (DAB) substrate (Vector Laboratories, Newark, CA) incubation for up to 10 min was performed to develop the color. Once the desired color development was achieved, the slides were rinsed in distilled water to stop any further color development. The developed slides were briefly counterstained with hematoxylin for 5 s and mounted with a medium (Richard‐Allan Scientific, Fisher Scientific). All quantitative analyses of the slides were performed using QuPath (University of Edinburgh) and ImageJ software.

### Toxicology Studies

The oxygen saturation (SpO_2_) of survival study porcine subjects was measured at baseline, immediately after embolization, and 14 days after embolization. ACT, complete blood count (CBC), and serum biochemical analyses were performed using collected blood samples. ACT was analyzed using the Celite ACT cartridge (Abbott Laboratories) and the i‐STAT handheld blood analyzer (Abbott Laboratories). CBC was analyzed using a veterinary hematology analyzer (Heska, Loveland, CO) and serum biochemical analysis was performed using a DRI‐CHEM 4000 chemistry analyzer (Heska).

### Evaluation of P‐LE in Porcine Microvascular Hemorrhage Models

Prior to inducing acute hemorrhage, a 2.8 F microcatheter was positioned at the desired location within the hepatic or splenic artery branches via percutaneous femoral artery ultrasound‐guided access. Initially, DSA was conducted to observe the baseline vasculature by injecting IOH (350 mg mL^−1^) through the catheter. To cause bleeding in the liver and stomach, Stiff guidewire (Boston Scientific, Marlborough, MA) was pushed through a microcatheter multiple times to induce vascular hemorrhage. To induce vascular hemorrhage in the renal tissue, under image guidance, an 18‐gauge, 15 cm long needle was percutaneously inserted to injure the peripheral parenchyma of the renal cortex where the blood vessels are typically sub‐mm. Angiography was performed to visualize contrast extravasation indicative of bleeding. Subsequently, the microcatheter was placed just proximal to the bleeding site, and P‐LE was injected until stasis was observed. A final angiography was performed to ensure hemostasis was achieved.

### Bacteria Susceptibility Test

The bacteria susceptibility test of P‐LE was performed using multiple drug‐resistant patient‐derived isolated pathogens (MRSA, VRE, CRE, *C. difficile*, and *C. auris*). These microbes were first diluted in 0.45% saline (Remel, San Diego, CA) to 0.5 McFarland absorbance. After mixing 100 µL of each microbe with 100 µL of serially diluted P‐LE, the mixture was incubated at 37 °C. Aerobic microbes (MRSA, VRE, CRE, and *C. auris*) were incubated for 24 h, and anaerobic microbe (*C. difficile*) was incubated for 48 h. Upon incubation, the mixes were plated directly onto sheep blood agar (Remel) using a cotton applicator (MEDLINE, Northfield, IL) without dilution. The plates were then further incubated at 37 °C to evaluate susceptibility. Susceptible concentrations: no growth; Intermediate concentrations: reduced growth compared to control; Resistance concentrations: No growth change compared to control.

### Statistical Analysis

Data are presented as mean ± SEM. Statistical analysis was performed by unpaired *t*‐test for 2 groups or one‐way ANOVA followed by Tukey's comparison test for multiple groups unless otherwise specified. All analyses were performed using GraphPad Prism 10. All P values are 2‐tailed, and P values less than or equal to 0.05 were considered statistically significant. ^*^ *p* < 0.05, ^**^ *p* < 0.01, ^***^ *p* < 0.001, ^****^ *p* < 0.0001. NS, no significance.

## Conflict of Interest

The authors declare no conflict of interest.

## Author Contributions

R.O. conceived the idea. R.O. and H.K. designed the experiments. H.K. and J.K. performed and analyzed the in vitro experiments. H.K. and Z.Z. performed the rat FA embolization studies. R.O. performed the canine and swine animal experiments. H.K., J.K., and, E.G., performed bacteria susceptibility tests. H.A. performed the CT analysis. H.K., J.K., and R.O. wrote the paper. R.O. is the principal investigator of the supporting grants.

## Supporting information

Supporting Information

Supplemental Video 1

Supplemental Video 2

## Data Availability

The data that support the findings of this study are available from the corresponding author upon reasonable request.
