# Supplementary material for: Biocompatible Liquid Embolic for the Treatment of Microvascular Hemorrhage
Source: Adv Sci (Weinh). 2024 Jul 25;11(34):2403615. doi: 10.1002/advs.202403615 (PMC11425845; doi:10.1002/advs.202403615)
Supplement: Supplementary file 1 — Supporting Information [file ADVS-11-2403615-s003.docx]

**Biocompatible liquid embolic for the treatment of microvascular hemorrhage**

*Hyeongseop Keum*^1^*, Jinjoo Kim*^1^*, Zefu Zhang*^1^*, Erin Graf*^2^*, Hassan Albadawi*^1,3^*, Yusuf M. Demirlenk*^1^*, and Rahmi Oklu*^1,3,^*

^1^The Laboratory for Patient-Inspired Engineering, Mayo Clinic, 13400 East Shea Blvd., Scottsdale, Arizona 85259, USA

^2^Department of Laboratory Medicine and Pathology, Mayo Clinic, 5777 E Mayo Blvd, Phoenix, Arizona 85054, USA

^3^Division of Vascular & Interventional Radiology, Mayo Clinic, 5777 E Mayo Blvd, Phoenix, Arizona 85054, USA

* Corresponding Author Email: Oklu.Rahmi@mayo.edu


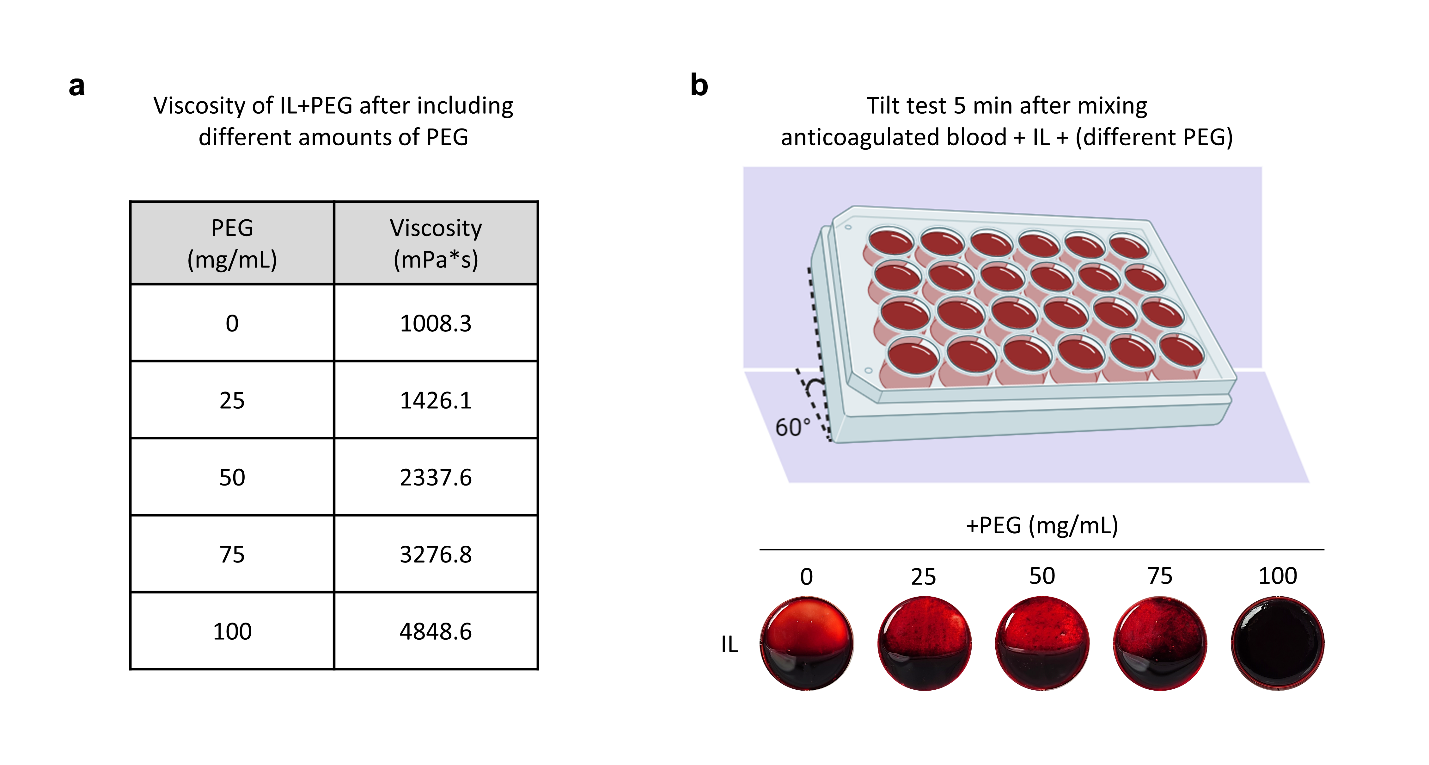


**Fig. S1.** **a,** Viscosity measurements of IL+PEG at different PEG concentrations. **b,** The tilt test was conducted 5 min after mixing various IL+PEG with anticoagulated blood to grossly assess the blood gelation. The results indicate that at a PEG concentration of 100 mg/mL, the gelation process was faster, with no signs of flow.


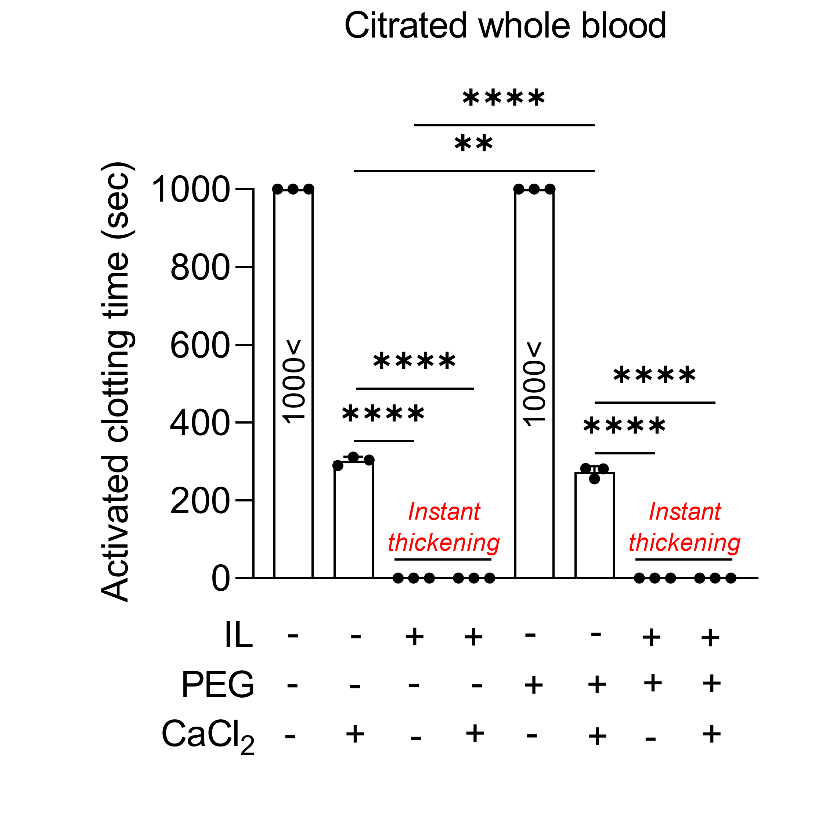


**Fig. S2.** Activated clotting time (ACT) of citrated whole blood when mixed with various permutations of IL (-/+), PEG (-/+), or CaCl_2_ (-/+) (n=3). Data are mean ± s.e.m.. Statistical analysis was performed using two-way ANOVA. ** *p* < 0.01 and **** *p* < 0.0001.


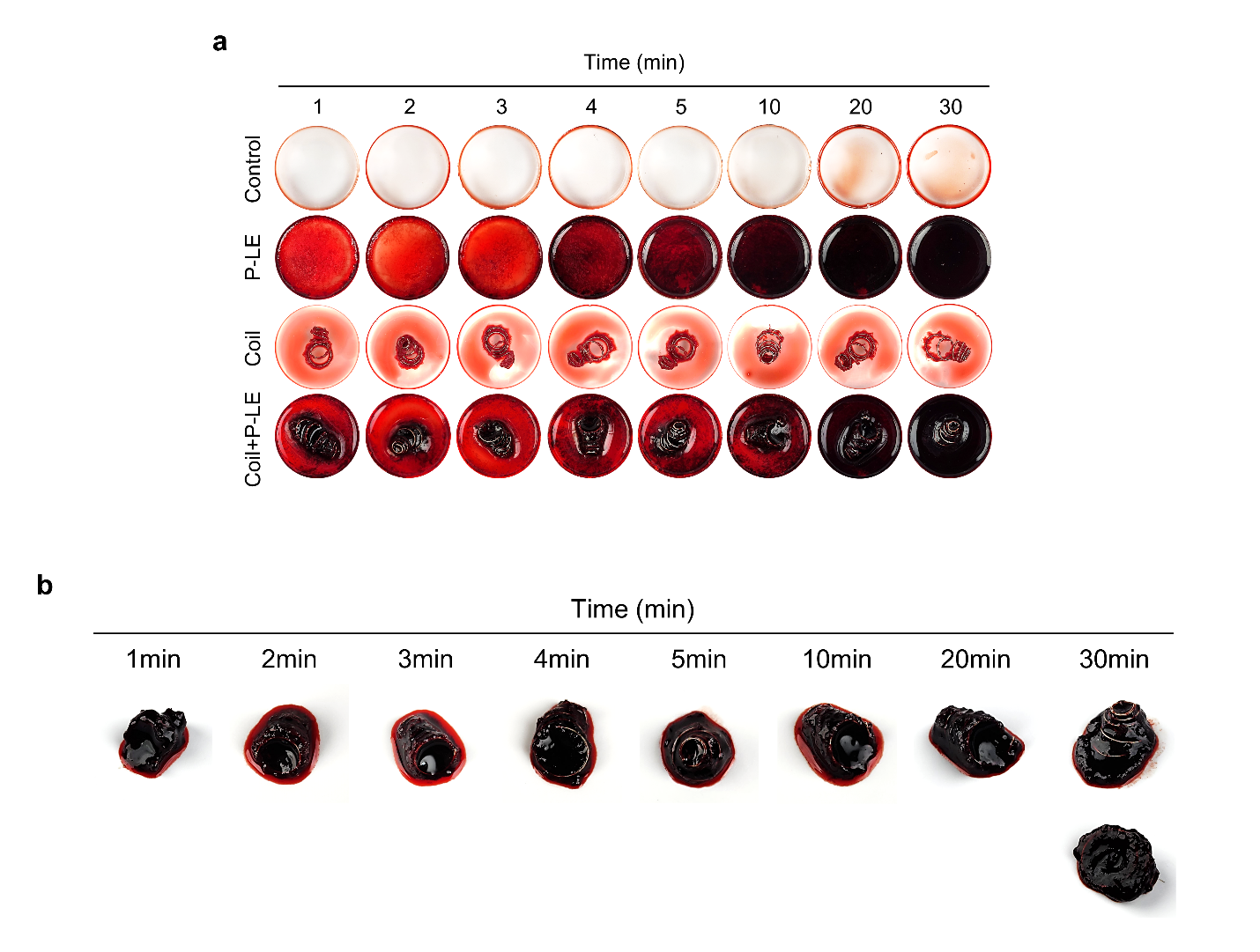


**Fig. S3. a,** Images of blood thrombosis test using anticoagulated citrated blood mixed with PBS (control), P-LE, Coil, or Coil+P-LE at 37℃ taken at different time points (1-30 min). **b,** Images showing the rapid formation of thrombus inside the coil when P-LE was mixed with anticoagulated blood suggesting the rescue of coil embolization in clinical scenarios.


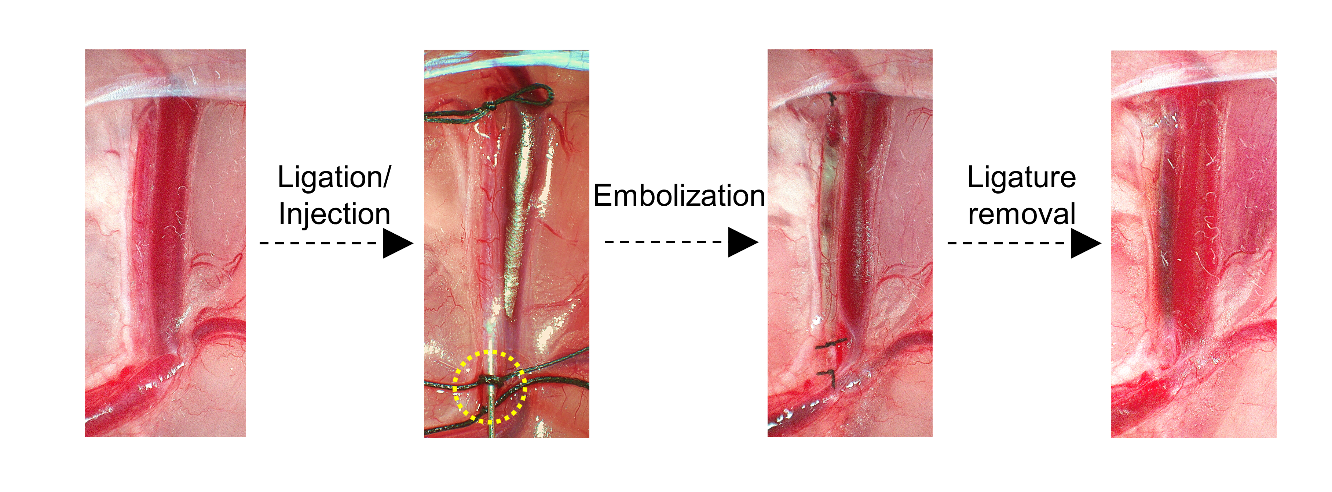


**Fig. S4.** Images showing the procedure of embolization in a rat femoral artery (FA). After ligation, materials were injected into the FA, filling the whole lumen. The proximal ligature was immediately removed to allow blood influx and mixing with P-LE. The distal ligature was subsequently removed to evaluate embolization and resulting hemostasis.


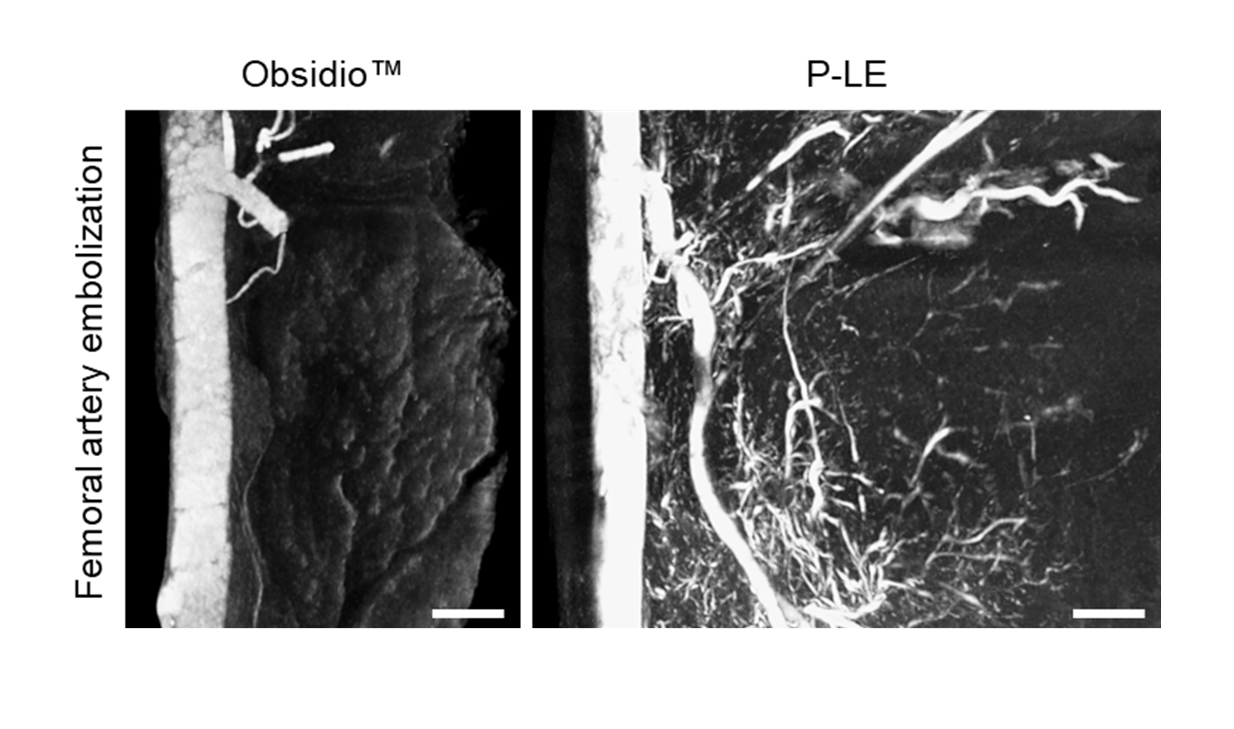


**Fig. S5.** The comparison between Obsidio™ and P-LE in a rat femoral artery embolization model. While Obsidio™ mostly remained upstream, P-LE readily reached and embolized sub-mm downstream vessels. Scale bars: 1 mm.


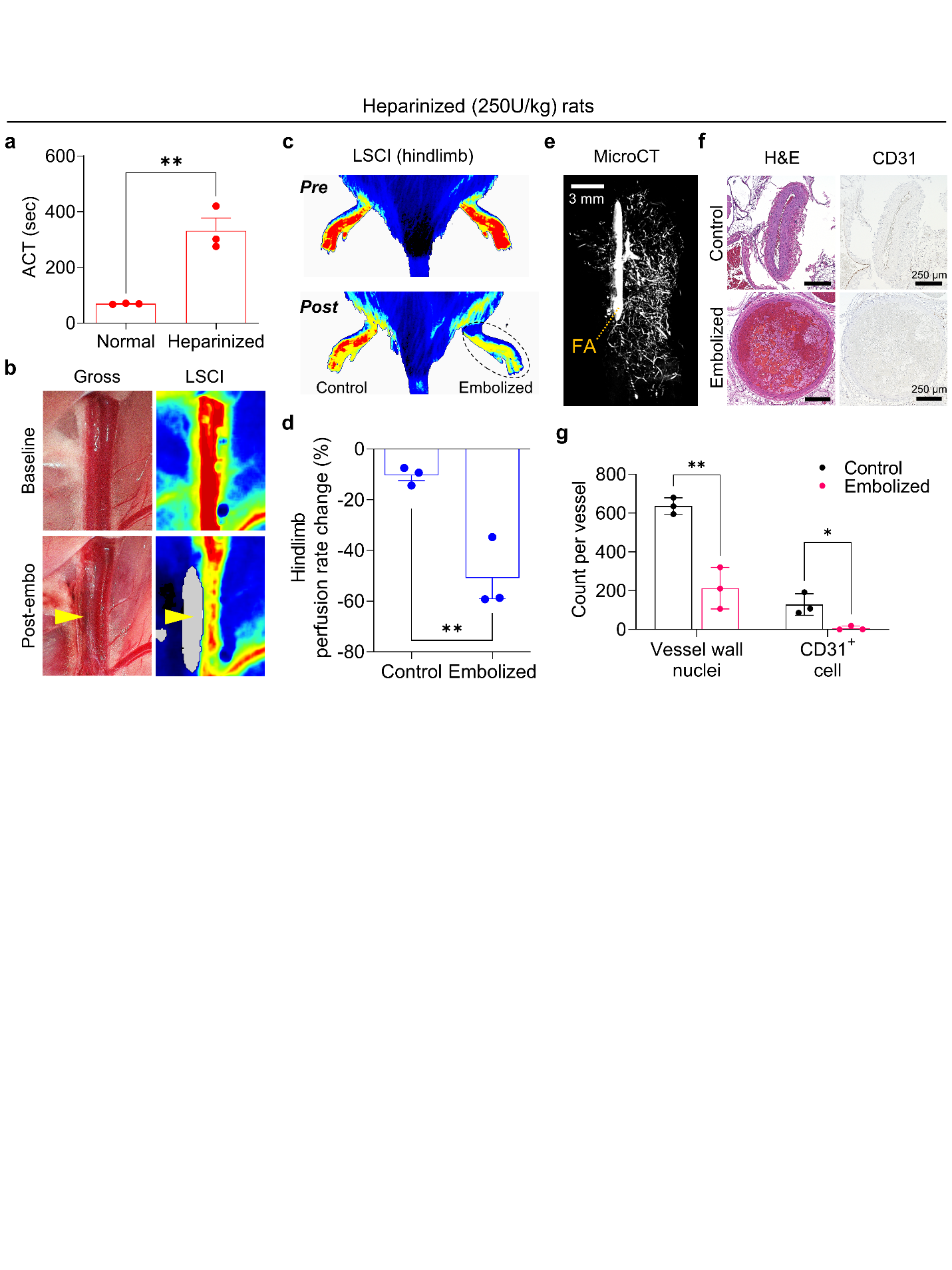


**Fig. S6. a,** ACT analysis of normal (pre-heparinization) blood and heparinized blood of rats (n=3). **b,** LSCI analysis of FA embolization in heparinized rats showing durable embolization with P-LE (yellow arrows indicate created embolus). **c,** LSCI images of control and embolized rat hindlimbs in a heparinized rat pre- and post-embolization with P-LE. **d,** Hindlimb perfusion rate change of control and embolized limbs compared to baseline in heparinized rats (n=3). **e,** Micro-CT image showing the embolization of FA and its downstream microvessels in a heparinized rat. **f,** Representative H&E and CD31 immunostaining images of non-embolized FA (control) and FA embolized with P-LE in a heparinized rat. **g,** Average count of vessel wall nuclei and CD31^+^ cells per vessel of control and embolized FA in heparinized rats (n=3). Data are mean ± s.e.m.. Statistical analysis was performed using an unpaired t-test in a and d, and multiple unpaired t-tests in g. * *p* <0.05 and ** *p* < 0.01.


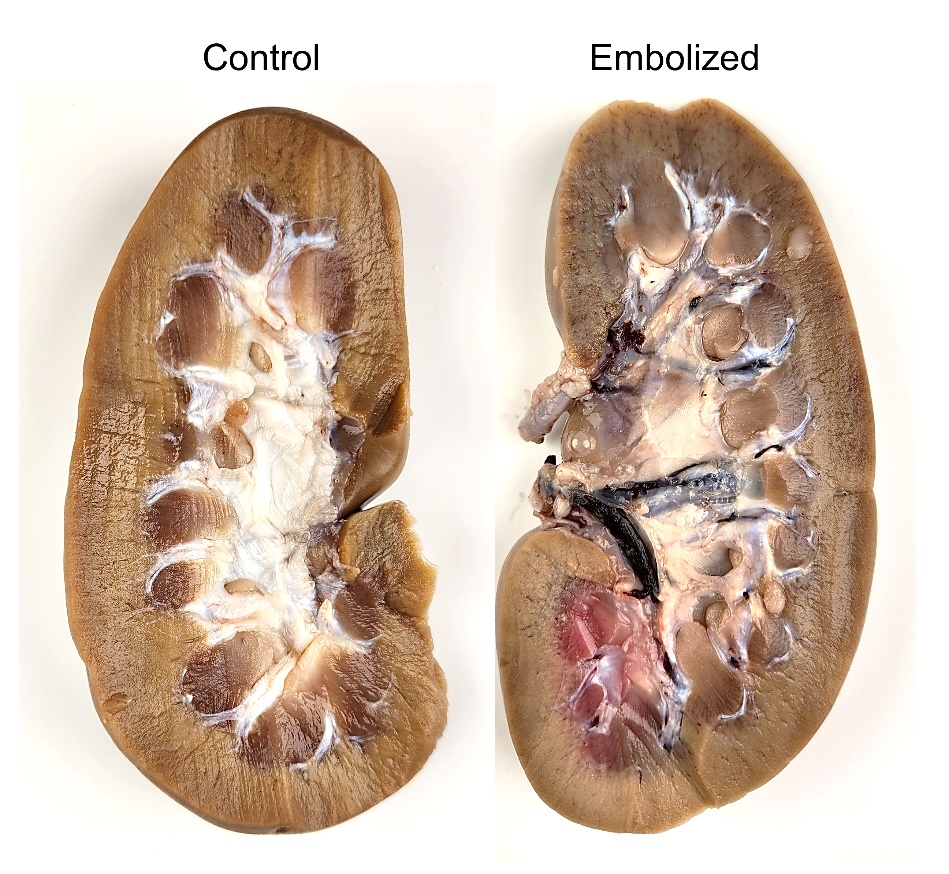


**Fig. S7.** A gross image of the respective bisected porcine kidney without embolization (control) and post-embolization (embolized).


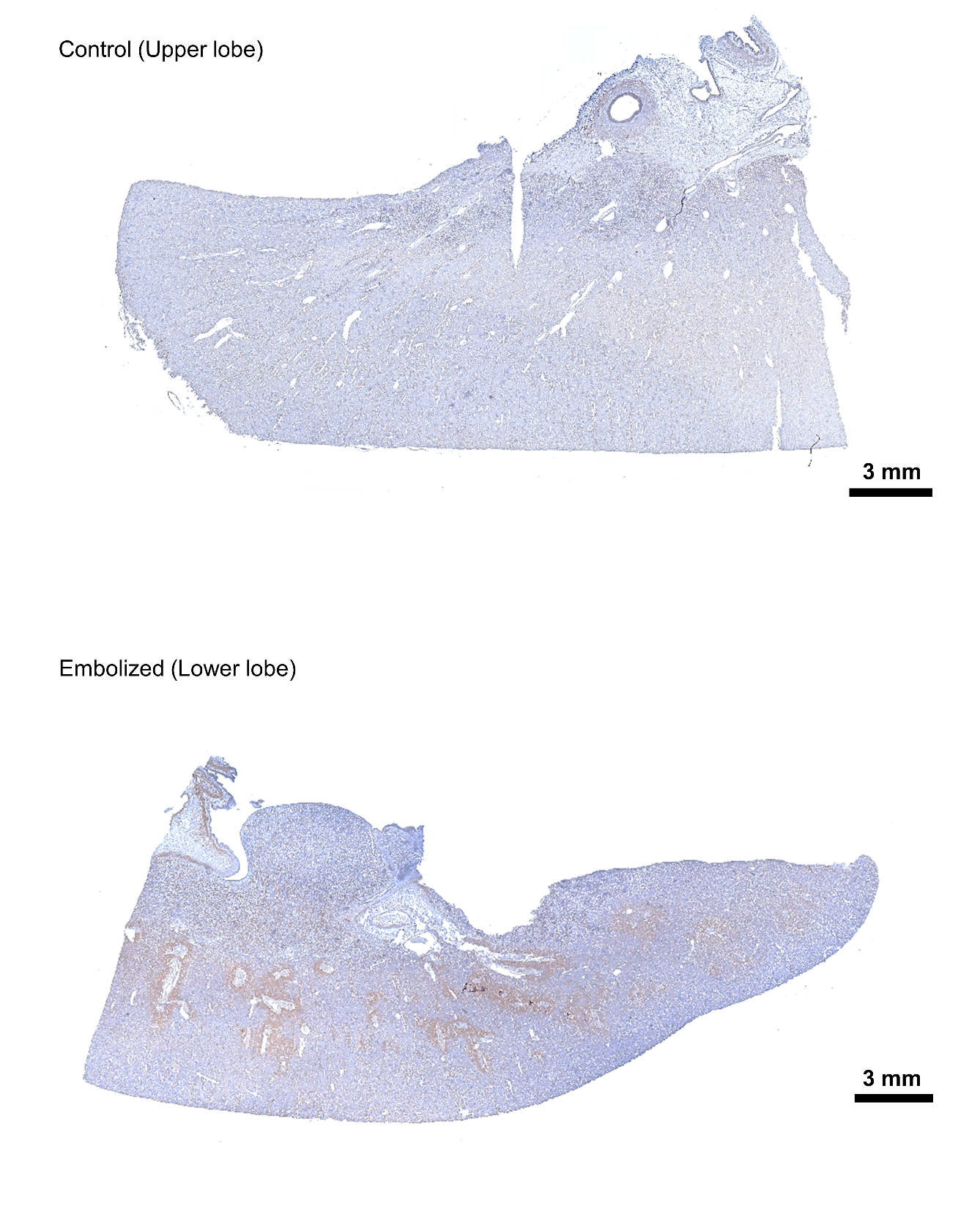


**Fig. S8.** HIF-2α immunostaining images of the upper lobe of a porcine kidney which was untreated (control) and the lower lobe which was embolized with P-LE (embolized) showing hypoxia regions surrounding the embolized vessels (brown regions).


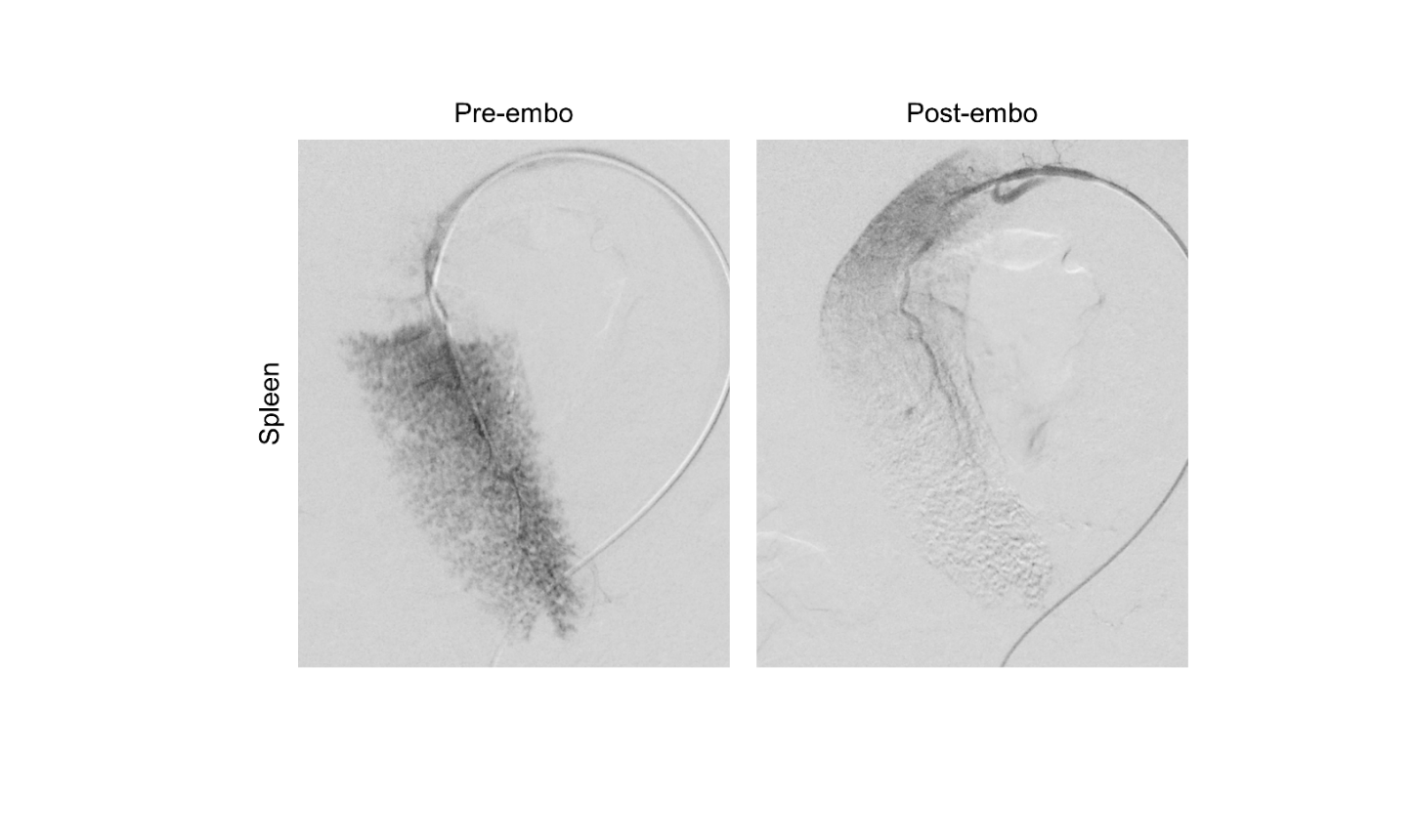


**Fig. S9.** Angiogram of spleen before and after embolization using P-LE. Post-embolization, the splenic sinusoids are no longer visible.


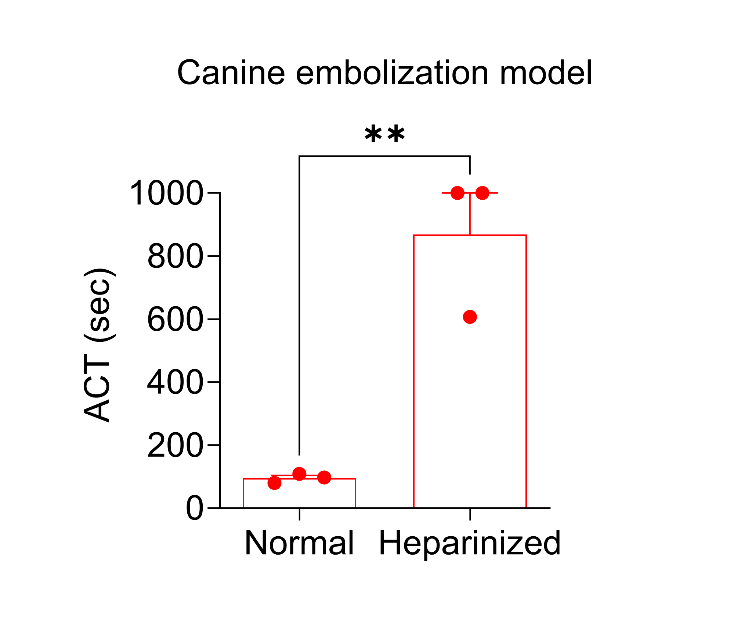


**Fig. S10.** ACT of normal (baseline) and heparinized canine whole blood showing successful anticoagulation with heparin (n=3). Data are mean ± s.e.m.. Statistical analysis was performed using an unpaired t-test in c. ** *p* < 0.01.


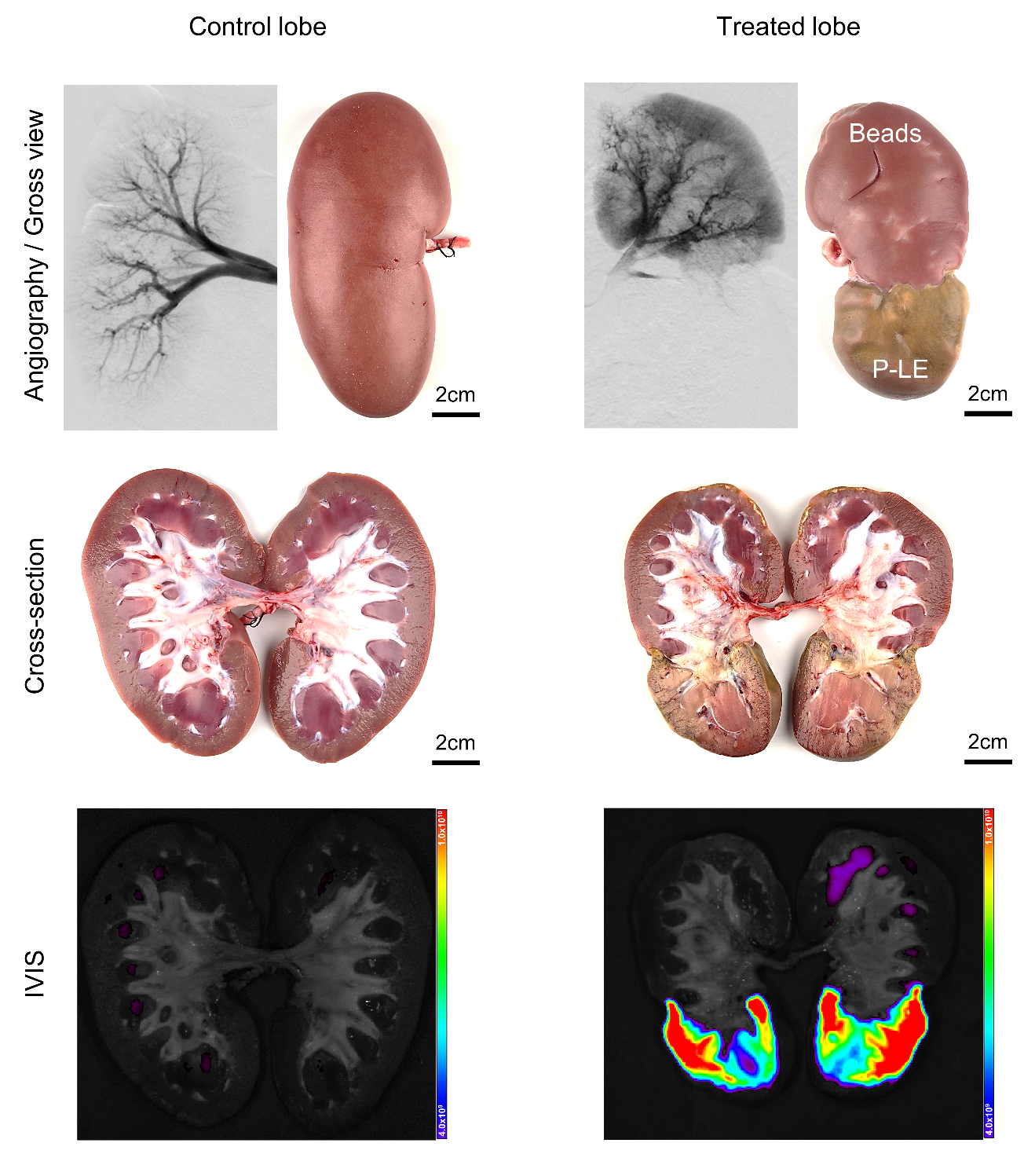


**Fig. S11.** DSA, gross images, cross-section images, and IVIS images of a non-embolized porcine kidney (control lobe) and an embolized porcine kidney (treated lobe). The upper pole was injected with microbeads and the lower pole was injected with P-LE. The only area injected with P-LE showed evident discoloration at D14 suggesting persistent embolization. IVIS imaging taken at D14 demonstrated intense ICG fluorescence while the pole injected with the bead barely retained the ICG signal suggesting a washout.


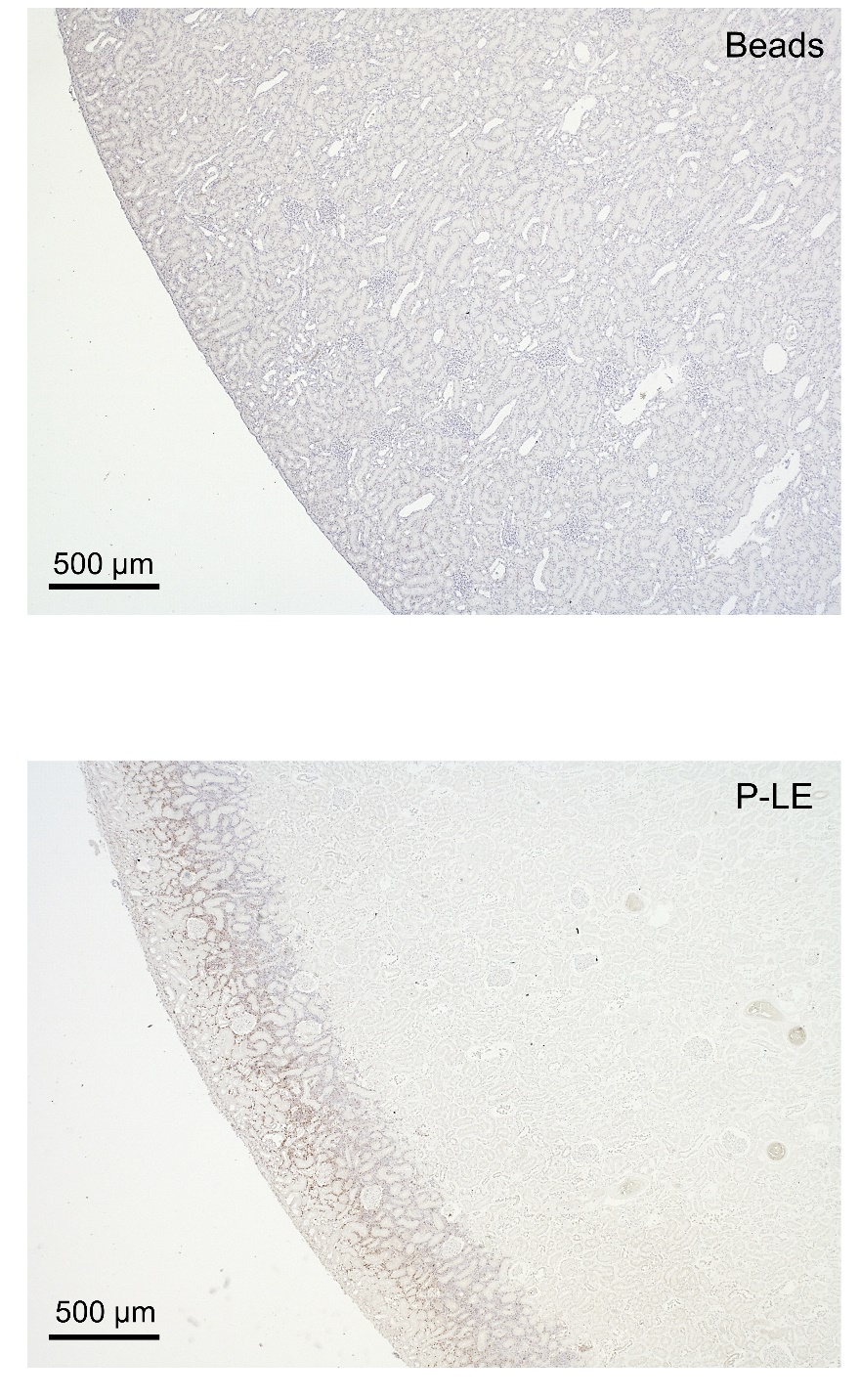


**Fig. S12.** MPO immunostaining of renal capsule of the tissues injected with beads and P-LE. Beads-injected tissue appears to be normal with cellularity and no sign of MPO. P-LE-injected tissue demonstrates no detectable nuclei in the parenchyma with infiltrated inflammatory cells only in the renal capsule region secondary to separate blood supply of the renal capsule from the capsular artery.


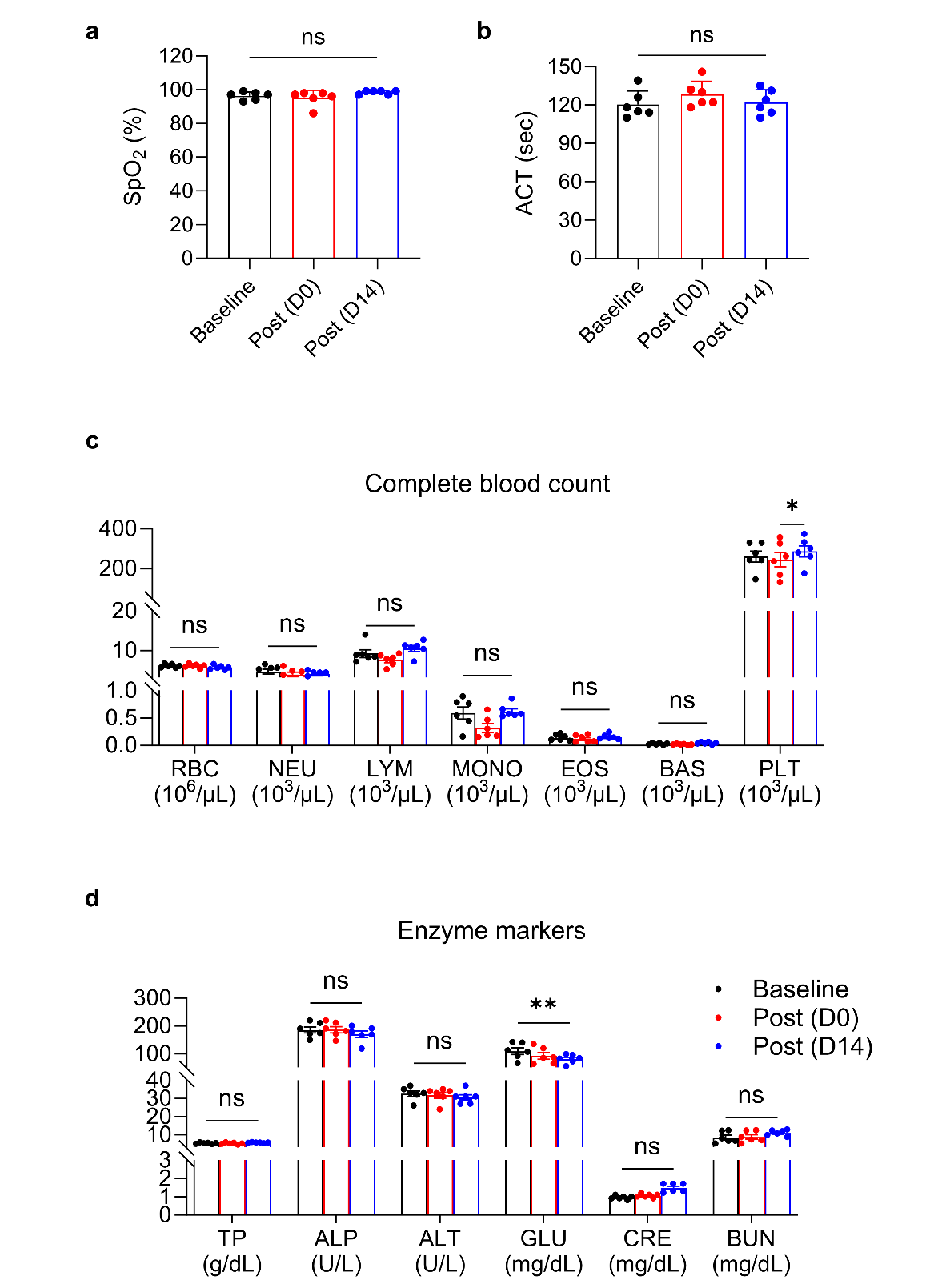


**Fig. S13. a-d,** Oxygen saturation (SpO2) (a), ACT (b), complete blood cell (CBC) counts (c), and serum biochemical analysis (d) results from baseline, D0 post-embolization, and D14 post-embolization blood samples in a survival porcine renal embolization model (n=6). Data are mean ± s.e.m.. Statistical analysis was performed using one-way ANOVA in a and b, and two-way ANOVA in c and d. ns, not significant; * *p* < 0.05 and ** *p* < 0.01.


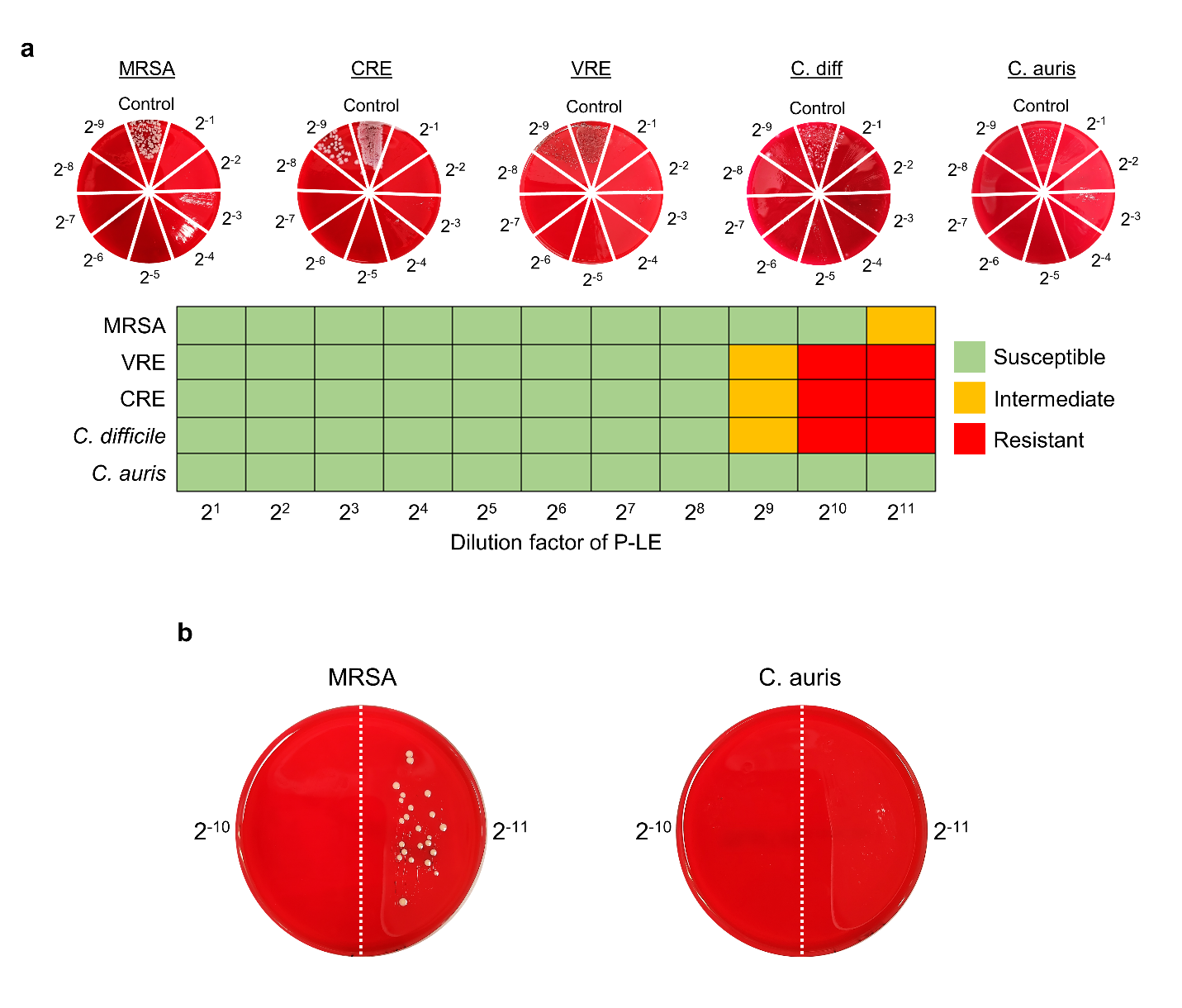


**Fig. S14. a,** Antimicrobial effect of P-LE against patient-derived antibiotic-resistant pathogens (*MRSA, CRE, VRE, C. diff, and C. auris*). P-LE was serially diluted with each microbe, incubated overnight, and inoculated on blood agar plates to examine the minimum inhibitory concentration of P-LE. The susceptibility for each tested microbe is summarized in the table. **b,** Antimicrobial effect of 2^10^ and 2^11^-fold diluted P-LE against MRSA and C. auris.
